# Comparison of subset selection methods in linear regression in the context of health-related quality of life and substance abuse in Russia

**DOI:** 10.1186/s12874-015-0066-2

**Published:** 2015-08-30

**Authors:** Olga Morozova, Olga Levina, Anneli Uusküla, Robert Heimer

**Affiliations:** 1 Department of Epidemiology of Microbial Diseases, Yale School of Public Health, New Haven, CT USA; 2 NGO Stellit, St. Petersburg, Russia; 3 Department of Public Health, University of Tartu, Tartu, Estonia

**Keywords:** Bayesian model selection, Penalized least squares, Stepwise regression, Linear regression, Subset selection, Quality of life, Substance abuse, HIV, Russia

## Abstract

**Background:**

Automatic stepwise subset selection methods in linear regression often perform poorly, both in terms of variable selection and estimation of coefficients and standard errors, especially when number of independent variables is large and multicollinearity is present. Yet, stepwise algorithms remain the dominant method in medical and epidemiological research.

**Methods:**

Performance of stepwise (backward elimination and forward selection algorithms using AIC, BIC, and Likelihood Ratio Test, *p* = 0.05 (LRT)) and alternative subset selection methods in linear regression, including Bayesian model averaging (BMA) and penalized regression (lasso, adaptive lasso, and adaptive elastic net) was investigated in a dataset from a cross-sectional study of drug users in St. Petersburg, Russia in 2012–2013. Dependent variable measured health-related quality of life, and independent correlates included 44 variables measuring demographics, behavioral, and structural factors.

**Results:**

In our case study all methods returned models of different size and composition varying from 41 to 11 variables. The percentage of significant variables among those selected in final model varied from 100 % to 27 %. Model selection with stepwise methods was highly unstable, with most (and all in case of backward elimination: BIC, forward selection: BIC, and backward elimination: LRT) of the selected variables being significant (95 % confidence interval for coefficient did not include zero). Adaptive elastic net demonstrated improved stability and more conservative estimates of coefficients and standard errors compared to stepwise. By incorporating model uncertainty into subset selection and estimation of coefficients and their standard deviations, BMA returned a parsimonious model with the most conservative results in terms of covariates significance.

**Conclusions:**

BMA and adaptive elastic net performed best in our analysis. Based on our results and previous theoretical studies the use of stepwise methods in medical and epidemiological research may be outperformed by alternative methods in cases such as ours. In situations of high uncertainty it is beneficial to apply different methodologically sound subset selection methods, and explore where their outputs do and do not agree. We recommend that researchers, at a minimum, should explore model uncertainty and stability as part of their analyses, and report these details in epidemiological papers.

**Electronic supplementary material:**

The online version of this article (doi:10.1186/s12874-015-0066-2) contains supplementary material, which is available to authorized users.

## Background

The problem of selecting a set of explanatory variables in regression modeling is well known and described in epidemiology and other disciplines [1–4]. The goal of the analysis, i.e. prediction, explanation, data mining, etc. determines the point of balance in the bias—variance tradeoff, where larger models generally reduce bias at the cost of increasing variance. In epidemiology, exploratory or hypothesis generating analysis aims to identify important correlates (or predictors) of the outcome in terms of clinical and statistical significance, and it normally involves subset selection techniques [5].

Automatic variable selection methods, including forward selection, backward elimination, and stepwise selection (hereafter, ‘stepwise methods’) [6, 7] were developed in 1960s and gained popularity in epidemiology and other fields for a number of reasons, including computational simplicity, relative ease of interpretation, and their implementation in major statistical software packages [3]. Stepwise methods became standard in epidemiology and remain so [8] despite a body of statistical and epidemiologic literature accumulated since early 1970s that provides theoretical and simulation evidence of their deficiencies [9–16].

Briefly, the main pitfalls of stepwise methods include: (a) the standard errors of the model coefficients are biased downward, and so are the p-values; (b) the absolute values of coefficients are biased upward; (c) the number of variables in the full model affects the number of noise variables in the final model; and (d) reliance on the single best model, while ignoring model uncertainty in producing the estimates [3, 9, 10].

A recent review determined that the most widely used variable selection methods in leading epidemiology journals were selection of variables based on prior knowledge and stepwise algorithms [8]. Stepwise algorithms still remain most widely used variable selection methods in epidemiology outside of genetics. A number of alternative variable selection methods have been proposed during the last couple of decades for a range of disciplines including epidemiology [17–19]. Model averaging methods allow parameter estimation that accounts for model uncertainty by averaging over all (or selected) models considered, and weighting each model by its likelihood [5]. Bayesian model averaging (BMA) incorporates prior knowledge about the covariates into the estimation procedure [20]. Penalized regression methods, including the lasso [21], the elastic net [22] and their extensions simultaneously select regression variables and estimate regression coefficients conditional on the selected penalization parameter(s). Although none of these methods is ideal, they enrich the epidemiologists’ methodological toolkit, and can offer better solutions in many situations.

In this paper we investigate the performance of traditional (stepwise regression using AIC, BIC, and the Likelihood Ratio Test) and some of the alternative (BMA, lasso, adaptive lasso, and adaptive elastic net) methods of subset selection in linear regression and making inferences about regression coefficients. We use a dataset from a cross-sectional survey conducted among people, who inject drugs (PWID) in St. Petersburg, Russia (*N* = 811). The dependent variable measures health-related quality of life (HRQoL), and the set of independent covariates includes 44 variables measuring demographics, behavioral, and structural factors. Identification of correlates of HRQoL is a vital problem in epidemiology, and is especially challenging in marginalized populations, such as PWID. A more parsimonious model (compared to the full model) would be beneficial in generating hypotheses about important correlates (and potentially predictors) of HRQoL, and would eventually yield valuable insights into applied problems of targeted interventions design in this population.

Analysis of the study data is particularly suitable for testing different subset selection methods, since the dataset includes a large number of potentially correlated variables, many of which are expected to have a decent explanatory power for the outcome of interest. While no general conclusions about the performance of different approaches can be drawn based on a single case study, our analysis provides useful insights into the problem of subset selection by applying various methods to the real world dataset. In this case study we explore properties of subset selection methods and demonstrate how they influence final model composition and the size of confidence intervals of regression coefficients, emphasizing the importance of careful choice of a proper method. Finally, for this case study we recommend BMA and adaptive elastic net as preferred methods. We argue that in situations of high uncertainty about the final model composition, the conservative approach would be to employ various robust methods and assign the degree of confidence to variables depending on how many methods selected a particular variable in the final subset.

## Methods

### Dataset

We used data from a cross-sectional study conducted among active PWID in St. Petersburg, Russia and Kohtla-Järve, Estonia. Participants were recruited using respondent driven sampling [23]. For the current analysis, we used the data from St. Petersburg site collected between November 2012 and June 2013. In our analysis we used unadjusted sample estimators, since adjusted inverse-probability weighting estimators have shown poor performance in simulation and empirical studies [24, 25].

The structured questionnaire included the following sections: demographics; contact with healthcare system and prison; alcohol, tobacco and drug use; injecting and sexual HIV risk practices; knowledge about HIV, tuberculosis and viral hepatitis; overdose; physical and psychological health; HIV and PWID disclosure and stigma (see Additional file 1: Study questionnaire (selected questions). As part of the study all participants were tested for HIV with the OraQuick Advance® HIV-1/2 rapid antibody test (OraSure, Bethleham, PA, USA).

### Ethics statement

Approval was obtained from the Institution Review Boards at Yale University and NGO Stellit in St. Petersburg.

### Dependent variable and independent correlates

The dependent variable is a measure of self-perceived health-related quality of life assessed using the visual analogue scale (VAS) of the EuroQoL 5D [26]. This is an integer-valued measure that varies from 0 (the worst imaginable state) to 100 (the best imaginable state).

The list of independent variables included basic demographic characteristics, history of drug use and drug abuse treatment, severity of alcohol use, severity of mental health problems, unsafe injecting and sexual behavior, awareness about the infection with HIV, viral hepatitis, tuberculosis and history of related treatment, experiences during interaction with health care system and police, PWID status disclosure, and drug use stigma (see Additional file 2: Variable codes). Alcohol use problems were assessed using CAGE scale with two or more positive responses being indicative of problematic use [27]. Mental health condition was assessed with MHI-5 scale, which ranges from 0 to 100, and a cut-off point of 52 was used [28]. The PWID disclosure scale consisted of seven questions (measured on 5-point Likert scale) [29]. We used two separate measures of disclosure: (a) to family or friends, and (b) to a healthcare provider. Internalized stigma scale [30] and stigma consciousness scale [31] were each a six items questionnaire (measured on 5-point Likert scale).

All covariates in the full model were selected *a priori* based on assumptions that they could reasonably be correlated with the outcome.

### Statistical analysis

Statistical analysis was performed in R Statistical Software (Foundation for Statistical Computing, Vienna, Austria). We used the package “MASS” to perform stepwise regression [32]; packages “glmnet” [33] and “gcdnet” [34] for penalized regression methods, and package “BMS” for Bayesian Model Averaging [35].

### Bivariate associations and full multivariate regression

Bivariate associations between all 44 correlates were estimated using ordinary least squares (OLS) regression. Full multivariate OLS regression included 40 correlates (totaling to 48 dummy variables). Four variables included in the bivariate analysis were excluded from multivariate regression and further methods due to complete collinearity with other variables in the model. 95 % confidence intervals (CIs) for coefficients in bivariate and full multivariate regression were estimated using the bootstrap (as an agnostic estimation method that does not rely on model form assumptions) [36, 37]. Number of bootstrap iterations = 2,000.

### Stepwise regression

For the conventional stepwise algorithms we employed backward elimination (BE) and forward selection (FS) strategies using hypothesis testing approach: Likelihood ratio test (LRT) (*p* = 0.05), and information theory criteria: AIC [38] and BIC [39]. A stepwise selection algorithm that combined BE and FS gave almost the same results as forward selection procedure. 95 % CIs for coefficients were estimated based on asymptotic sampling distribution of the final fitted model and using bootstrap method (number of iterations = 2,000). To follow the conventional method, the main analysis used the asymptotic 95 % CIs, and those based on bootstrap are presented for comparison in Additional file 3.

We used bootstrap to assess the stability of subsets selected with BE and FS strategies for each of the three criteria [40] (number iterations = 2,000). Since penalized regression methods and BMA treat each dummy variable of multi-level categorical variables separately, for comparability purposes, we used the same approach when exploring stability of stepwise regression.

### Penalized regression

The general idea of penalized regression is that the loss function (usually squared error loss) is minimized under a constraint that penalizes for model complexity and/or large absolute values of coefficients [36].

Ridge regression uses the L2 penalty (sum of squares of regression coefficients multiplied by the penalty factor), thus shrinking regression coefficients closer to zero [36]. It deals well with highly correlated variables, but does not perform variables selection. Lasso [21] uses L1 penalty (sum of absolute values of regression coefficients multiplied by the penalty factor), thus allowing for simultaneous variable selection (by forcing some of the coefficients to be exactly zero) and coefficient estimation. Elastic net [22] combines L1 and L2 penalties with separate penalty factors, thus allowing for subset selection with a better performance in the presence of multicollinearity. The modification of lasso, called adaptive lasso [41] uses different L1 penalty factors for every covariate in regression model, and a similar modification for elastic net, called adaptive elastic net, was developed [42].

All penalized regression methods require selection of the regularization parameter (hereafter, λ), which determines the strength of the imposed penalty. The most commonly used method to select an optimal value of λ is cross-validation (CV) [36, 43]. Usually two values of λ are considered: the value that minimizes the CV mean squared error (MSE) (denoted as λ_min_), and the maximum value within one standard error from λ_min_ (denoted as λ_1se_).

We performed variable selection using lasso, adaptive lasso, and adaptive elastic net. To run adaptive lasso we used penalty factor weights based on coefficients estimated via ridge regression [41], and the same L1 penalty factor weights were used in adaptive elastic net. The regularization parameter for L2 penalty was determined by running the conventional elastic net with the following penalty: [α × L1 penalty + (1-α) × L2 penalty], where α = 0.5; and using λ that minimized the CV MSE. In all penalized regression methods regularization parameter was selected using 10-fold CV, and results are reported for λ_min_ and λ_1se_.

Estimation of standard errors for lasso coefficients is an area of active research. We estimated standard errors using bootstrap (number of iterations = 2,000) with fixed regularization parameter(s) and fixed penalty weights (for adaptive lasso and adaptive elastic net) [21]. This method allows estimation of standard errors for non-zero coefficients of lasso and its extensions.

As with stepwise regression, we assessed the stability of selected subsets using bootstrap (number of iterations = 2,000). In all methods values of λ_min_ and λ_1se_ were re-calculated in every bootstrap iteration. Adaptive lasso and adaptive elastic net penalty weights were also re-calculated in every bootstrap iteration.

### Bayesian model averaging

Comprehensive overviews of Bayesian methods and the BMA methodology have been published [20, 44–46]. Briefly, BMA methods estimate regression coefficients based on posterior probabilities of all (or *m* best) models considered. In the case of relatively low number of variables (less than 20), all possible subsets are enumerated and evaluated, while in the situation of a very large number of variables, model space search algorithms are usually employed [20]. Depending on the subject knowledge, one can specify prior probabilities or use non-informative priors. BMA algorithms estimate posterior model probabilities, inclusion probabilities for each variable, regression coefficients and their standard deviations.

In our analysis we used non-informative uniform priors and the MCMC algorithm [20] to search the model space. Variable inclusion probabilities, and values of regression coefficients and standard deviations were estimated: (a) based on aggregate information from sampling chain with posterior model distributions based on MCMC frequencies (hereafter, “aggregate information”); (b) based on 100 best (highest posterior probability) models from the sampling chain with posterior model distributions based on exact marginal likelihoods (hereafter, “100 best models”). If a particular evaluated model did not include a particular variable, the corresponding coefficient value was considered 0 for the purpose of estimation of regression coefficients and standard deviations. For subset selection we used median inclusion probability model (corresponding to 0.5 posterior inclusion probability threshold) [47].

## Results

The characteristics of study subjects along with regression coefficients and 95 % CIs for bivariate and full multivariate regressions are presented in Table 1. The dependent variable of interest—a EuroQoL 5D VAS measure of the health related quality of life—has a mean value of 63.97 (standard deviation = 15.34), and a median value of 65.00 (range is 5–100). Summary information on the distribution of dependent variable is presented in the Additional file 4. Correlation matrix of regression covariates is presented in the Additional file 5.

**Table 1:** Characteristics of study participants; bivariate and full multivariate linear regressions^a^, (*N* = 811)

Independent variables	n (%)^b^	Bivariate OLS regression		Full multivariate OLS regression (*N* = 803)^c^	
		Beta	95 % CI	Beta	95 % CI
I. SOCIO-DEMOGRAPHIC CHARACTERISTICS					
Sex:					
Male	631 (77.8)	Ref	-	Ref	-
Female	180 (22.2)	1.72	(−0.62 ; 4.06)	−3.25	(−5.61 ; −0.88)
Age (median = 32 y.o):					
Less than 32 y.o.	397 (49.0)	Ref	-	Ref	-
32 y.o. or older	414 (51.0)	−5.21	(−7.27 ; −3.14)	−4.81	(−6.69 ; −2.93)
Education:					
Primary or basic	62 (7.6)	Ref	-	Ref	-
Secondary, vocational or at least some higher	749 (92.4)	7.37	(2.89 ; 11.84)	3.54	(−0.61 ; 7.68)
Main source of income:					
Legal source	677 (83.5)	Ref	-	Ref	-
Illegal source	134 (16.5)	−3.27	(−5.78 ; −0.76)	−0.35	(−2.75 ; 2.05)
Level of income:					
Coping well	245 (30.2)	Ref	-	Ref	-
Coping is difficult (or very difficult)	566 (69.8)	−8.99	(−11.18 ; −6.80)	−3.86	(−6.00 ; −1.73)
Living arrangements:					
Someone else’s house	274 (33.8)	Ref	-	Ref	-
Owned or rented place	512 (63.1)	−3.24	(−5.35 ; −1.13)	1.73	(−0.33 ; 3.79)
Shelter/no fixed place	25 (3.1)	−5.00	(−10.80 ; 0.81)	0.97	(−4.74 ; 6.69)
Marital status:^d^					
Not married	554 (68.4)	Ref	-	Ref	-
Married	256 (31.6)	−0.21	(−2.55 ; 2.13)	−0.96	(−3.40 ; 1.49)
II. ALCOHOL AND DRUG USE					
Alcohol abuse using CAGE scale:					
CAGE = 0–1	274 (33.9)	Ref	-	Ref	-
CAGE = 2-4	534 (66.1)	−10.10	(−12.20 ; −8.00)	−1.76	(−4.00 ; 0.47)
Age of first drug use (cannabis excluded; median = 16 y.o.):					
17 y.o. or older	280 (34.5)	Ref	-	Ref	-
16 y.o. or younger	531 (65.5)	−6.64	(−8.70 ; −4.58)	−3.38	(−5.47 ; −1.30)
Main drug of use:					
(Meth)-amphetamines	27 (3.3)	Ref	-	Ref	-
Methadone/Fentanyl	221 (27.3)	−6.92	(−11.80 ; −2.04)	1.43	(−3.51 ; 6.37)
Heroin	563 (69.4)	−14.74	(−19.39 ; −10.08)	0.87	(−4.01 ; 5.74)
Poly-drug use in the last 4 weeks:^e^					
Injected 1 class of drugs	697 (85.9)	Ref	-	Ref	-
Injected 2 or more classes of drugs	114 (14.1)	−6.15	(−9.08 ; −3.22)	−3.62	(−6.38 ; −0.87)
Frequency of injecting drugs (days during the last 4 weeks; median = 20):					
19 days or less	337 (41.6)	Ref	-	Ref	-
20 days or more	474 (58.4)	−8.37	(−10.49 ; −6.25)	−1.39	(−3.88 ; 1.09)
Frequency of injecting drugs (times per day; median = 1):					
One	437 (54.0)	Ref	-	Ref	-
Two or more	372 (46.0)	−7.69	(−9.79 ; −5.60)	−0.40	(−2.61 ; 1.80)
Used non-sterile injecting equipment at least once in the last 4 weeks:					
No (or don’t know)	344 (42.4)	Ref	-	Ref	-
Yes	467 (57.6)	−10.54	(−12.59 ; −8.50)	−2.05	(−4.41 ; 0.30)
Ever used non-sterile injecting equipment:					
No	79 (9.7)	Ref	-	Ref	-
Yes	732 (90.3)	−10.94	(−14.08 ; −7.81)	−0.55	(−4.13 ; 3.04)
Getting sterile injecting equipment (any unused syringes in last 4 weeks):					
No	46 (5.7)	Ref	-	Ref	-
Yes	765 (94.3)	9.12	(4.77 ; 13.48)	−0.97	(−5.14 ; 3.20)
Ever overdosed:					
No	284 (35.0)	Ref	-	Ref	-
Yes	527 (65.0)	−6.58	(−8.75 ; −4.42)	0.10	(−2.12 ; 2.32)
III. MENTAL HEALTH					
Mental health problems score:					
Lower score on mental health problems	427 (52.7)	Ref	-	Ref	-
Higher score on mental health problems	384 (47.3)	−9.27	(−11.28 ; −7.26)	−2.61	(−4.85 ; −0.38)
IV. SEXUAL RISK					
Sexually active in the last 6 months:					
No	188 (23.2)	Ref	-	Ref	-
Yes	623 (76.8)	1.75	(−0.73 ; 4.23)	0.91	(−1.69 ; 3.52)
Involved in sexual work in the last 6 months:					
No	757 (93.3)	Ref	-	Ref	-
Yes	54 (6.7)	−0.16	(−3.35 ; 3.03)	2.80	(−1.05 ; 6.66)
Paid for sex in the last 6 months:					
No	748 (92.2)	Ref	-	Ref	-
Yes	63 (7.8)	7.80	(4.65 ; 10.95)	2.25	(−1.11 ; 5.60)
Condom use during the last sexual intercourse:					
Yes	378 (46.6)	Ref	-	N/A	-
No	238 (29.3)	−0.79	(−3.29 ; 1.72)		
Don’t know	195 (24.0)	−2.44	(−5.11 ; 0.24)		
HIV and Hepatitis C status of primary sexual partner:					
HIV and HCV negative or unknown	155 (19.1)	Ref	-	Ref	-
Known to be HIV or HCV positive	218 (26.9)	−11.09	(−14.14 ; −8.04)	−1.98	(−5.05 ; 1.09)
No primary partner in the last 6 months	438 (54.0)	−8.23	(−11.00 ; −5.47)	−2.02	(−5.04 ; 1.00)
V. INFECTIOUS DISEASES HISTORY AND STATUS					
Ever been tested for HIV:					
No (or don’t know)	52 (6.4)	Ref	-	Ref	-
Yes	759 (93.6)	−1.95	(−6.04 ; 2.13)	3.63	(−0.58 ; 7.84)
HIV status awareness:					
Result of the most recent HIV test is negative, unknown or never tested	428 (52.8)	Ref	-	N/A	-
Result of the most recent HIV test is positive	383 (47.2)	−9.71	(−11.74 ; −7.68)		
HIV status (based on study testing):					
Negative	359 (44.3)	Ref	-	Ref	-
Positive	452 (55.7)	−9.20	(−11.26 ; −7.15)	−2.71	(−6.55 ; 1.13)
Receiving regular HIV care:					
HIV-negative or unaware	428 (52.8)	Ref	-	Ref	-
HIV+; receives regular HIV care	125 (15.4)	−0.87	(−3.41 ; 1.67)	1.46	(−3.05 ; 5.98)
HIV+; does not receive regular HIV care	258 (31.8)	−13.77	(−15.95 ; −11.59)	−4.32	(−8.36 ; −0.28)
Tuberculosis history awareness:					
No (or don’t know)	757 (93.3)	Ref	-	Ref	-
Yes	54 (6.7)	−7.46	(−11.15 ; −3.76)	−3.32	(−6.43 ; −0.22)
Hepatitis C history awareness:					
No	126 (15.5)	Ref	-	N/A	-
Yes	685 (84.5)	−10.13	(−13.02 ; −7.23)		
Treatment of Hepatitis C:					
Never diagnosed with HCV	126 (15.5)	Ref	-	Ref	-
HCV+, never been offered treatment	591 (72.9)	−11.09	(−14.00 ; −8.17)	−4.56	(−7.62 ; −1.50)
HCV+, was offered treatment, but did not receive it	50 (6.2)	−7.45	(−12.58 ; −2.32)	−7.37	(−12.75 ; −1.99)
HCV+, was offered treatment and received it	44 (5.4)	−0.27	(−4.53 ; 3.98)	−2.02	(−6.80 ; 2.76)
Hepatitis B history awareness:					
No	401 (49.4)	Ref	-	Ref	-
Yes	410 (50.6)	−9.31	(−11.29 ; −7.34)	−0.05	(−2.36 ; 2.25)
Ever vaccinated against Hepatitis B:					
No (or don’t know)	525 (64.7)	Ref	-	Ref	-
Yes (at least one dose)	286 (35.3)	7.29	(5.07 ; 9.50)	1.63	(−0.74 ;4.00)
VI. CONTACT WITH TREATMENT SERVICES AND PRISON					
History of incarceration:					
No	537 (66.2)	Ref	-	Ref	-
Yes	274 (33.8)	−2.41	(−4.64 ; −0.18)	−1.49	(−3.40 ; 0.42)
Having basic medical insurance:					
No	156 (19.3)	Ref	-	Ref	-
Yes	654 (80.7)	2.39	(−0.40 ; 5.17)	1.46	(−1.06 ; 3.98)
Receiving any healthcare services in the last 12 months:					
Received	546 (67.3)	Ref	-	Ref	-
Not received	265 (32.7)	−3.00	(−5.32 ; −0.68)	−1.46	(−3.56 ; 0.64)
Ever received drug abuse treatment:					
No	229 (28.2)	Ref	-	N/A	-
Yes	582 (71.8)	−5.79	(−8.15 ; −3.43)		
Receiving detoxification services in the last 6 months:					
Did not need detox services	646 (79.7)	Ref	-	Ref	-
Needed, but did not receive detox	99 (12.2)	−10.74	(−14.12 ; −7.35)	−5.39	(−9.00 ; −1.78)
Needed and received detox	66 (8.1)	−4.00	(−8.04 ; 0.04)	−4.82	(−7.96 ; −1.67)
Ever had difficulties obtaining drug abuse treatment:					
Never received treatment (or don’t know)	233 (28.7)	Ref	-	Ref	-
Had no difficulties	482 (59.4)	−4.44	(−6.89 ; −1.99)	1.26	(−0.93 ; 3.45)
Had difficulties	96 (11.8)	−10.56	(−13.92 ; −7.20)	−0.74	(−4.14 ; 2.66)
Had difficulties obtaining medical care because of drug use:					
No (or don’t know)	764 (94.2)	Ref	-	Ref	-
Yes	47 (5.8)	−5.10	(−9.48 ; −0.73)	−4.22	(−8.21 ; −0.22)
VII. STIGMA, DISCLOSURE AND POLICE HARASSMENT					
Ever experienced police confiscate syringes:					
No	599 (73.9)	Ref	-	Ref	-
Yes	212 (26.1)	−6.00	(−8.41 ; −3.59)	−0.48	(−3.02 ; 2.06)
PWID status disclosure to family/friends:^f^					
Rather disclosed	420 (51.8)	Ref	-	Ref	-
Rather did not disclose	391 (48.2)	−7.06	(−9.10 ; −5.01)	−0.65	(−2.78 ; 1.48)
PWID status disclosure to a healthcare provider:^g^					
Rather disclosed	278 (34.3)	Ref	-	Ref	-
Rather did not disclose	533 (65.7)	3.83	(1.67 ; 5.99)	−0.16	(−2.51 ; 2.19)
Internalized PWID stigma:^h^					
Low	417 (51.4)	Ref	-	Ref	-
High	394 (48.6)	−9.14	(−11.15 ; −7.13)	−3.68	(−5.92 ; −1.44)
PWID stigma consciousness:^h^					
Low	343(42.3)	Ref	-	Ref	-
High	468 (57.7)	−1.68	(−3.81 ; 0.45)	1.52	(−0.52 ; 3.56)

*95 % CI* 95 % confidence interval, *HRQoL* Health-related quality of life, *OLS* Ordinary Least Squares, *PWID* people who inject drugs, *Ref* Reference Category, *VAS* Visual Analogue Scale

^a^Dependent Variable is EuroQoL 5D VAS measure of the HRQoL

^b^Numbers may not sum up to total due to missing values, and % may not sum up to 100 due to rounding

^c^The adjusted R^2^ = 0.37. Four variables (Condom use during the last sexual intercourse, HIV status awareness, Hepatitis C awareness, Ever received drug abuse treatment) were not included into the multivariate regression, because of complete collinearity with other variables in the model

^d^Married = legally married or living as married; Not married = widowed, divorced or never married

^e^The following classes of drugs are included: opiates, amphetamines, and cocaine

^f^Based on five questions, each measured on 5-point Likert scale. Individual items scores were summed and dichotomized by median

^g^Based on one question measured on 5-point Likert scale, and dichotomized by median

^h^Both internalized stigma scale and stigma consciousness scale are six items questionnaires measured on the 5-point Likert scale. Individual items scores were summed and dichotomized by median

**Fig. 1:**
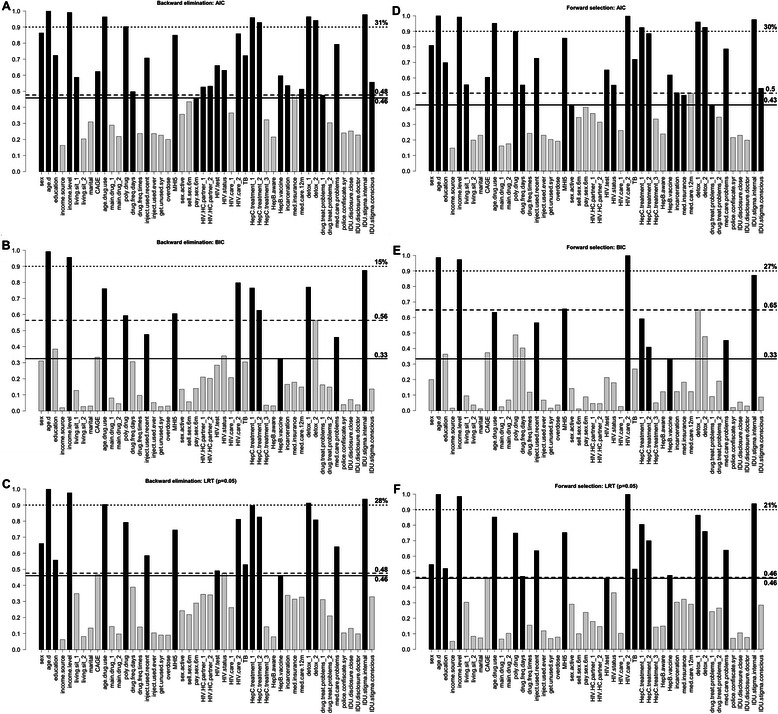
Bootstrap frequency of covariates selection in the final model using stepwise algorithms. Dependent variable is EuroQoL 5D visual analogue scale measure of the health-related quality of life. **a** shows results of backward elimination regression using AIC, **b**—using BIC, and **c**—using Likelihood Ratio Test (*p* = 0.05). **d**, **e** and **f** show results of forward selection regression with AIC, BIC and LRT (*p* = 0.05) correspondingly. Black bars represent variables selected in the final model, and light grey bars—variables excluded from the final model. Solid line and the number next to it correspond to the minimum frequency among variables included in the final model; dashed line and the number next to it correspond to the maximum frequency among variables excluded from final subset. Dotted line corresponds to the frequency = 0.9, and number next to it shows the percentage of variables in the final model with inclusion frequency over 0.9 (out of the number of variables selected in the final model). Description of variable names is provided in the Additional file 2

The regression coefficients along with their 95 % CIs estimated asymptotically and with bootstrap for the models selected using BE and FS techniques with AIC, BIC and the LRT (*p* = 0.05) are presented in the Additional file 3. BE and FS algorithms resulted in very similar models, while models differed substantially depending on the inclusion criterion used. The number of variables retained in the model with AIC is 29 (of which 14 are significant at 0.05 level) for BE method, and 27 (16 are significant) for FS method. Stepwise regression with BIC resulted in models that included 13 and 11 variables for BE and FS correspondingly (all selected variables are significant). When LRT (*p* = 0.05) was used the algorithms retained 18 and 19 variables in the final model for BE and FS correspondingly (with 18 being significant in both cases). Figure 1 presents the results of the model stability evaluation using bootstrap. In all cases except FS: LRT (*p* = 0.05) the highest inclusion frequency among non-selected variables was bigger than the lowest inclusion frequency among selected variables, and in case of FS: LRT (*p* = 0.05) these frequencies were equal. When AIC or LRT (*p* = 0.05) were used as model selection criteria, the differences in mentioned inclusion probabilities were relatively small ranging from 0 to 0.07. In the case of BIC, however, the highest inclusion frequency among non-selected variables was substantially bigger than the lowest inclusion frequency among selected variables, being 0.23 and 0.32 for BE and FS correspondingly. The percentage of variables with inclusion frequency over 0.9 (of the number of variables in the final model) ranged from 15 % (BE: BIC) to 31 % (BE: AIC) (Fig. 1).

**Fig. 2:**
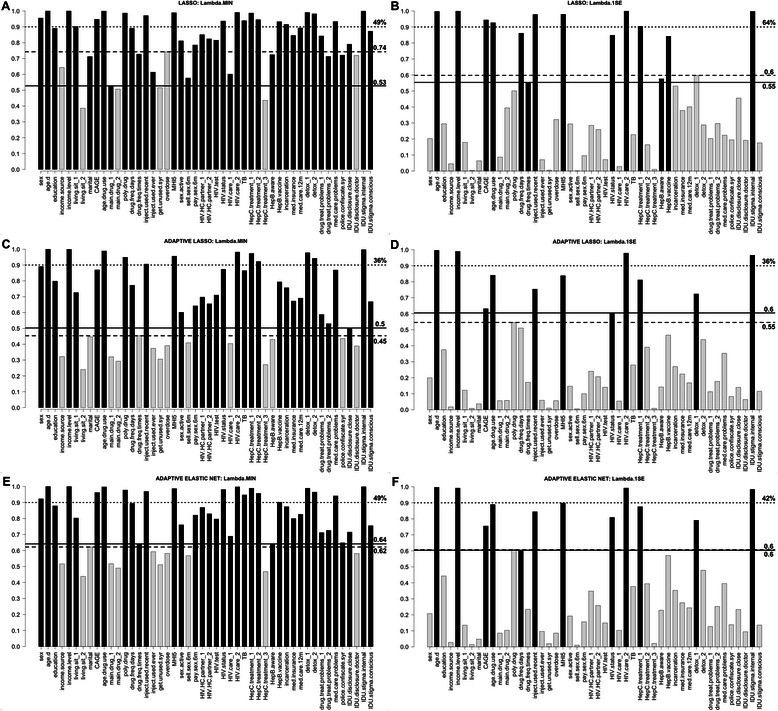
Bootstrap frequency of covariates selection in the final model using penalized regression. Dependent variable is EuroQoL 5D visual analogue scale measure of the health-related quality of life. **a** shows results of lasso corresponding to λ_min_, **b**—lasso corresponding to λ_1se_ ; **c** and **d**—adaptive lasso with λ_min_ (**c**) and λ_1se_ (**d**); and **e** and **f**—adaptive elastic net with λ_min_ (**e**) and λ_1se_ (**f**). Black bars represent variables selected in the final model, and light grey bars—variables excluded from the final model. Solid line and the number next to it correspond to the minimum frequency among variables included in the final model; dashed line and the number next to it correspond to the maximum frequency among variables excluded from final subset. Dotted line corresponds to the frequency = 0.9, and number next to it shows the percentage of variables in the final model with inclusion frequency over 0.9 (out of the number of variables selected in the final model). Description of variable names is provided in the Additional file 2

Detailed outputs of lasso, adaptive lasso, and adaptive elastic net regressions are presented in the Additional file 6, and include regularization paths, graphs of CV results, estimates of regression coefficients along with 95 % CIs, and the bootstrap inclusion frequencies for variables. For lasso the highest inclusion frequency among non-selected variables was bigger than the lowest inclusion frequency among selected variables (both for λ_min_ and λ_1se_). In adaptive lasso and adaptive elastic net, however, the situation was opposite—inclusion frequencies of selected model variables were bigger than those of non-selected in all cases except adaptive elastic net λ_1se_, where the corresponding frequencies were equal (Fig. 2). Adaptive lasso demonstrated better stability in terms of the difference between the lowest selected and the highest non-selected variables inclusion frequency. On the other hand, lasso and adaptive elastic net demonstrated better performance in terms of percentage of model variables with inclusion frequency over 0.9 (Fig. 2). The number of significant variables was generally smaller in all penalized regression methods compared to stepwise methods (Fig. 4).

**Fig. 3:**
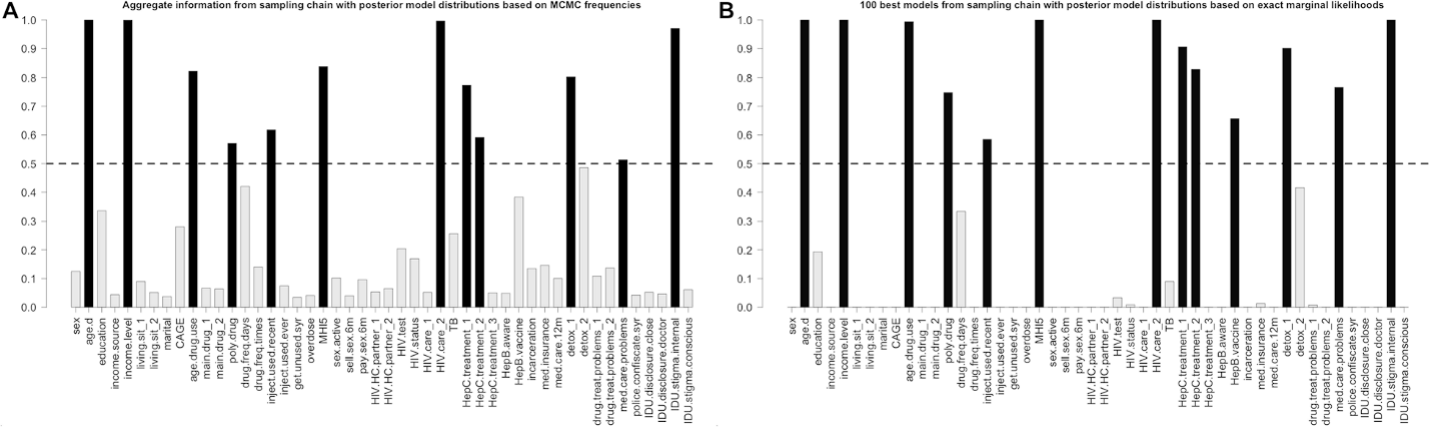
Bayesian model averaging: posterior inclusion probabilities of independent variables in linear regression. Dependent variable is EuroQoL 5D visual analogue scale measure of the health-related quality of life. **a** shows covariates posterior inclusion probabilities (PIP) based on aggregate information from sampling chain with posterior model distribution based on MCMC frequencies. **b** shows covariates PIP based on 100 best models from sampling chain with posterior model distributions based on exact marginal likelihoods. Dashed line corresponds to the subset selection PIP threshold, which equals 0.5 (median inclusion probability model). Description of variable names is provided in the Additional file 2

**Fig. 4:**
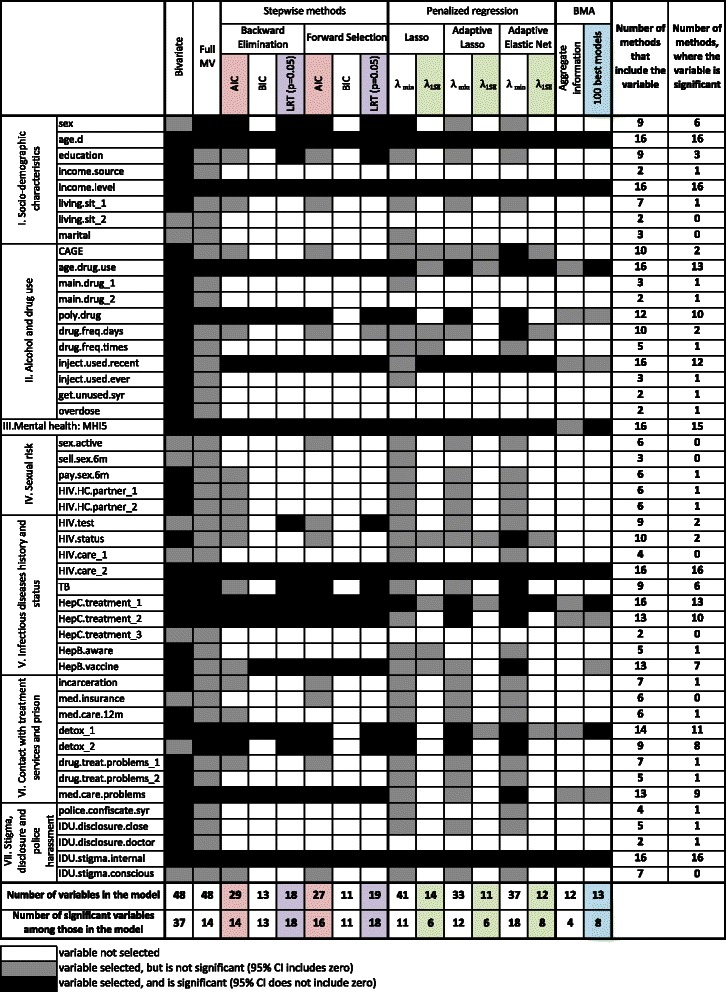
Summary of the resulting linear regression models obtained with different subset selection methods. Dependent variable is EuroQoL 5D visual analogue scale measure of the health-related quality of life. 95 % CI, 95 % Confidence/Credible interval; Full MV, full multivariate regression; HRQoL, health-related quality of life. Description of variable names is provided in the Additional file 2

Figure 3 presents posterior inclusion probabilities for each variable from the BMA analysis. Subsets selected based on aggregate information and 100 best models were very similar (the latter subset included one additional variable). The model size was 12 and 13 correspondingly. In aggregate information model 95 % credible intervals of 4 out of 12 variables did not include zero, and in 100 best subset model it was 8 out of 13 (Fig. 4). BMA posterior inclusion probabilities for model variables, along with regression coefficients and 95 % credible intervals, as well as graphs presenting information regarding the sampling process and posterior distribution of model size are presented in the Additional file 7.

Figure 4 presents the summary and comparison of the variable selection methods used in this analysis. It shows the inclusion and significance in the final subset for every variable for every method used in the analysis. It provides summary of the model size and number of significant variables by method, as well as subset inclusion summary by variable. Out of 48 variables analyzed, 8 were included into the final model by all variable selection methods. 7 were not selected by either of methods (besides bivariate and full multivariate regression).

Correlates of the worse health-related quality of life selected most often included: older age, lower income level, younger age of the drug use initiation, using non-sterile injecting equipment during the last 4 weeks, having higher mental health problems score, being HIV positive and not getting regular HIV care (compared to being HIV negative or unaware), being Hepatitis C positive and never being offered treatment (compared to being Hepatitis C negative or unaware), and having high internalized drug use stigma.

Table 2 provides the summary of performance of the analyzed methods in terms of model stability, whether model uncertainty is properly incorporated in the statistical inference procedure, as well as computational efficiency of the algorithms.

**Table 2:** Summary of methods performance

Method	Stability of model selection	Incorporating model uncertainty	Computational efficiency (running time)^a^
I. STEPWISE REGRESSION METHODS			
Backward elimination (AIC)	Moderate	Do not incorporate model uncertainty in the estimation of regression coefficients and standard errors.	Model selection: 5.4 s
			Estimation of SE with bootstrap^b^: 30.9 s
Backward elimination (BIC)	Very poor		Model selection: 5.6 s
			Estimation of SE with bootstrap^b^: 15.0 s
Backward elimination (LRT)	Moderate		Model selection: 5.1 s
			Estimation of SE with bootstrap^b^: 19.2 s
Forward selection (AIC)	Moderate		Model selection: 2.8 s
			Estimation of SE with bootstrap^b^: 28.5 s
Forward selection (BIC)	Very poor		Model selection: 1.9 s
			Estimation of SE with bootstrap^b^: 13.8 s
Forward selection (LRT)	Moderate		Model selection: 3.1 s
			Estimation of SE with bootstrap^b^: 19.8 s
II. PENALIZED REGRESSION METHODS			
Lasso	Poor (λ_min_)	Model uncertainty is partially incorporated into the estimation and inference procedure via λ tuning step, and estimation of standard errors using bootstrap.	Lasso algorithm: 0.02 s
	Good (λ_1se_)		10-fold CV: 0.5 s
			Estimation of SE with bootstrap^b^: 394.0 s
Adaptive lasso	Good (λ_min_)		Estimation of weights (ridge regression): 1.6 s
	Good (λ_1se_)		Adaptive lasso algorithm: 0.02 s
			10-fold CV: 0.5 s
			Estimation of SE with bootstrap^b^: 411.2 s
Adaptive elastic net	Good (λ_min_)		Estimation of weights (ridge regression): 1.6 s
	Good (λ_1se_)		Estimation of λ for L2 penalty (elastic net): 1.2 s
			Adaptive elastic net algorithm: 0.2 s
			10-fold CV: 1.4 s
			Estimation of SE with bootstrap^b^: 3,265.3 s
III. BAYESIAN MODEL AVERAGING			
Bayesian model averaging (using MCMC to search model space)	PIPs of regression covariates inform model selection. Bootstrap gave selection frequencies that were almost identical to PIPs (data not shown).	Model uncertainty is properly incorporated into the estimation of regression coefficients and their standard deviations (provided that MCMC chain converged and the algorithms managed to search the entire model space).	250.8 s
			(1,000,000 iterations, chain converged)

*AIC* Akaike Information Criterion, *BIC* Bayesian Information Criterion, *CV* cross-validation, *LRT* Likelihood Ratio Test, *MCMC* Markov Chain Monte Carlo, *PIP* posterior inclusion probability, *SE* standard error

^a^The analysis is run on a 1.7 GHz Intel(R) Core(TM) i5 processor with 4.00 GB of DDR3 memory

^b^In all cases of estimation of standard errors using bootstrap number of iterations = 2,000

## Discussion

In this paper we present a case study that compares the outputs of conventional (stepwise) and alternative (penalized regression, BMA) subset selection methods in linear regression. This is a valuable real-world example, common in epidemiology, of the challenges of subset selection when (i) the number of candidate covariates is large, (ii) most of candidate variables have good explanatory power for the outcome of interest, and (iii) multicollinearity is present. Previous theoretical work and simulation studies have shown that conventional stepwise algorithms perform particularly poorly when thus challenged [2, 9], and are outperformed by BMA [11, 48] and penalized regression methods [49, 50].

Some statisticians, however, argue that disadvantages of stepwise methods are over-emphasized [51]. Studies that report agreement between stepwise and alternative variable selection methods [52] may mislead the reader to the conclusion of their similar performance. While it is not unlikely to observe agreement in subset selection methods in the situation of relatively few potentially weakly correlated variables, the deficiencies of stepwise methods become more apparent when number of candidate variables increase and issues of multicollinearity come into play. As such, the salient illustration of the importance of careful choice of the subset selection method is the huge variability in the model size and composition derived with different methods in our analysis (Fig. 4). The final model size varies from 41 (lasso, λ_min_) to 11 (adaptive lasso, λ_1se_ and FS: BIC), and the percentage of significant variables among those selected to enter final model varies from 100 % (BE: BIC, FS: BIC, and BE: LRT) to 27 % (lasso, λ_min_).

The most widely used subset selection method—stepwise regression with LRT (*p* = 0.05) returns a model of size 18 (BE) and 19 (FS), where 18 covariates are significant (in both cases), illustrating the failure to incorporate model uncertainty into the statistical inference procedure. Stepwise BIC selects models of size 13 (BE) and 11 (FS), since BIC generally favors smaller models by imposing a larger model size penalty. While stepwise BIC provides a desired parsimony (with large sample size), the selected models are highly unstable (Fig. 1), and suffer from the same problem of underestimated standard errors. Compared to BIC, stepwise regression with AIC performs better in terms of model selection stability (Fig. 1). The improved stability is likely a result of a less severe model size penalty, which indirectly alleviates the problem of multicollinearity. The stepwise AIC, however, provides little improvement compared to LRT as it still fails to account for model uncertainty and treats a selected model as if it was pre-specified, thus returning biased standard errors [53].

Penalized regression methods provide flexible model selection tools. By specifying the form of the penalty term (L1, L2, or their combination), and by selecting the strength of the penalty (the value of regularization parameter λ), the researcher can control the model size and address the problem of multicollinearity. There are theoretical challenges in estimation of standard errors in lasso and its extensions, however use of the bootstrap with fixed λ allows estimating the confidence intervals for coefficients that are not shrunk to zero conditional on the selected value of λ [21]. The advantages of lasso are mostly notable in situations when the number of covariates is close to or bigger than number of observations. This explains its wide use in genetic studies. Several drawbacks of lasso have been noted, including unsatisfactory performance in the presence of multicollinearity [54]. Elastic net regression improves the performance of lasso in this respect [22], and assigning weights equal to 0.5 to L1 and L2 penalty tends to select or exclude groups of correlated variables together [55]. The use of a single unique regularization parameter can lead to selection of irrelevant variables and over-shrink large coefficients of important correlates. Adaptive lasso [41] reduces estimation bias and improves stability by putting individual penalty on every regression variable. Adaptive elastic net [42] demonstrated good performance in dealing with multicollinearity, estimation bias and model selection stability.

In our analysis all penalized regression methods select models of relatively large size (between 33 and 41) for λ_min_ (Fig. 4). This is explained by the fact that most of the covariates have some explanatory power, and the sample size is large compared to the number of covariates; thus the cross-validation with MSE loss favors bigger models. When we drew a random sample of 80 observations from the dataset, we obtained a model size of 9, 5 and 9 for lasso, adaptive lasso and adaptive elastic net, respectively, for λ_min_. Given such property, the use of λ_1se_ is a more sensible option in our example. It provides the desired parsimony, while the percentage of deviance explained reduces moderately (from 0.40 for λ_min_ to 0.32 for λ_1se_ in all three methods). The model size obtained through penalized regression methods corresponding to λ_1se_ (14 for lasso, 11 for adaptive lasso, and 12 for adaptive elastic net) is comparable to that of BIC stepwise (13 and 11 variables). All three penalized regression methods, however, demonstrate a substantial improvement in stability compared to stepwise BIC (Figs. 1 and 2, Table 2). Further, unlike in stepwise regression, the tuning of λ with CV and the estimation of standard errors using the bootstrap offer an improvement in addressing the issue of model uncertainty. Thus the number of significant correlates in penalized regression methods with λ_1se_ is substantially smaller than in stepwise BIC (Fig. 4, Table 2).

BMA offers an improvement compared to conventional methods by directly incorporating model uncertainty into the process of model selection, and estimation of regression coefficients and their standard deviations (Table 2). Model ranking by the marginal likelihood provides information regarding the model uncertainty, and posterior inclusion probabilities of covariates offer an intuitive and convenient aid to subset selection. BMA is primarily a method for estimation of regression coefficients and their standard deviations. While making proper inferences is itself highly valuable, the BMA algorithm includes estimation of likelihoods of different models, and can therefore return the highest likelihood model (often called best subset). An alternative approach, which we used in this paper, suggests using the median probability model as a subset selection method [47]. It performs subset selection based on posterior inclusion probabilities of covariates, setting a threshold at the level of 0.5.

BMA is not free of problems, of which the most important are specification of priors and dealing with computationally intractable number of candidate models [20]. In our analysis we chose conservative non-informative uniform priors that did not take a full advantage of the power of Bayesian approach. In situations when an investigator has more information about the model covariates before performing the data analysis, specification of informative priors is desirable, however the choice should be justified. With the advancement of technology, BMA is now implemented in many statistical software packages allowing for its wider use [35, 56]. Software packages that implement BMA normally include several versions of the model space search algorithms, of which MCMC is the most widely used. Computational efficiency can be an issue for the implementation of BMA, because the MCMC chain has to converge, and convergence can be hard to reach, especially in the big data context (Table 2). Moreover, with MCMC one can never be fully confident that the algorithm has successfully searched the entire model space. One of the common ways to address this problem is to run the chain with different starting models and compare the results. Obtaining similar results increases the confidence that an entire model space was properly searched, but doesn’t offer a guarantee.

In our analysis we present two ways of posterior inclusion probability estimation: based on aggregate information from all models based on MCMC frequencies, and based on exact marginal likelihoods of 100 best models. These two methods result in very similar selected subsets that differ by one (of 48) variable. Since it is desirable to incorporate full model uncertainly, which is already limited by non-complete enumeration of models, it is advised to use posterior inclusion probabilities based on aggregate information. In our example this approach, consistent with expectation, returns a parsimonious model consisting of 12 variables, and the most conservative 95 % credible intervals of all methods, where only 4 variables are significant.

Several limitations of our analysis should be mentioned. In the absence of a gold standard for subset selection, and not knowing the data generating process, such as in cases of simulation studies, we had to rely on indirect measures of methods performance, such as investigation of model stability and assessment of how well the model uncertainty is addressed by different methods. This is an inherent limitation of using real-life data for analyses such as ours compared to simulation studies. However, this is also one of the strengths of this paper, since it offers an example of the real-life behavior of different methods that were extensively evaluated in simulation studies. All of the methods analyzed in this paper and the results of our analysis are only applicable to the linear regression. While the methods themselves can be extended to logistic regression, proportional hazard model, etc., the results we presented cannot be directly extrapolated to other types of regression models. Moreover, we assumed the form of the model (i.e. OLS regression), and only considered the uncertainty coming from its composition, while a broader model selection problem also includes considerations of the uncertainty regarding the functional form of the model. We have performed our case study using one dataset that represents a typical example of data used in epidemiological research among PWIDs. In our analysis we aimed to focus on analytical approaches and present very detailed outputs of the methods and their comparison in order for this example to serve as both the demonstration of approach to methods comparison, and the presentation of actual findings from such comparison. Extending similar analysis to multiple datasets might be one of the potential future research directions. From the practical perspective, we hope that our example would motivate investigators to employ similar strategies in applied data analysis.

Our analysis demonstrates that it is beneficial to apply different subset selection methods, and explore where their outputs do and do not agree (Fig. 4). This is especially useful in exploratory analysis, situations of high uncertainty about the correct model, and if one is interested in finding a set of the strongest correlates or predictors of the outcome, and wants to improve the credibility of findings. When interpreting the results, it is important to differentiate between the features of statistically sound methods that stem from varying tradeoff between bias and variance [5], and the deficiencies in selection, estimation, and inference inherent to methods that violate the principles of statistical theory [10]. This approach should not be confused, however, with using multiple methods, selecting one that gives the most ‘desired’ result, and presenting it as if it was the only one method deployed.

In our analysis all correlates that are selected most often using different methods make intuitive sense, and the findings are generally in line with other studies conducted in similar populations [57–60].

## Conclusions

Our analysis emphasizes the importance of understanding of the properties of various subset selection methods, and a careful choice of method (or a combination thereof) that would correspond to the goals of analysis. As we have shown, different subset selection methods return models with very different sizes and estimated coefficients. It is, therefore, vital that the researcher defend the reasons behind choosing a particular technique based on assumptions, theoretical considerations, research questions, and intended use of the results.

Based on performance of different methods in this case study and previous theoretical work, we discourage the use of stepwise algorithms, and recommend BMA that accounts for the full model uncertainty, and adaptive elastic net (with λ_1se_ when N is large) in cases such as ours. We also encourage researchers to explore model uncertainty and stability as part of their analyses, and report these details in epidemiological papers.

## Additional files

Additional file 1: **Study questionnaire (selected questions).** Study questions used to create variables for the analysis. (PDF 187 kb)
Additional file 2: **Variable codes.** Provides detailed description of variable codes used in the main text and other additional files. (PDF 60 kb)
Additional file 3: **Stepwise regression.** The regression coefficients along with their 95 % CIs, and the bootstrap inclusion frequencies of infependent variables for the models selected using automatic stepwise selection algorithm (backward elimination and forward selection) with AIC, BIC and the Likelihood ratio test (*p* = 0.05). (PDF 229 kb)
Additional file 4: **Distribution of the dependent variable.** Descriptive statistics, histogram and Q-Q plot of the dependent variable. (PDF 80 kb)
Additional file 5: **Correlation matrix.** Matrix of all pairwise correlation coefficients of independent variables considered in subset selection. (PDF 64 kb)
Additional file 6: **Penalized regression methods.** Detailed outputs of lasso, adaptive lasso, and adaptive elastic net regressions; includes regularization paths, graphs of cross validation results, estimates of regression coefficients along with 95 % CIs, and the bootstrap inclusion frequencies for independent variables. (PDF 920 kb)
Additional file 7: **Bayesian model averaging.** BMA posterior inclusion probabilities for model variables, along with regression coefficients and 95 % credible intervals for variables selected in the final model; includes graphs of posterior model size distribution, MCMC chain convergence, and variable inclusion in 2,000 models with the highest likelihood. (PDF 460 kb)

## Abbreviations

AIC: Akaike information criterion
BE: Backward elimination
BIC: Bayesian information criterion
BMA: Bayesian model averaging
CI: Confidence (or credible) interval
FS: Forward selection
HRQoL: Health-related quality of life
lasso: least absolute shrinkage and selection operator
LRT: Likelihood ratio test
MCMC: Markov Chain Monte Carlo
OLS: Ordinary least squares
PWID: People who inject drugs
VAS: Visual analogue scale

## References

[1] George EI (2000). The Variable Selection Problem. J Am Stat Assoc.

[2] Greenland S (1989). Modeling and variable selection in epidemiologic analysis. Am J Public Health.

[3] Rothman KJ, Greenland S, Lash TL (2008). Modern Epidemiology.

[4] Miller A (2002). Subset Selection in Regression.

[5] Burnham KP, Anderson DR (2002). Model Selection and Multimodel Inference: A Practical Information-Theoretic Approach.

[6] Efroymson MA, Ralston A, Wilf H (1960). Multiple regression analysis. Mathematical Methods for Digital Computers.

[7] Draper NR, Smith H (1998). Applied regression analysis.

[8] Walter S, Tiemeier H (2009). Variable selection: current practice in epidemiological studies. Eur J Epidemiol.

[9] Derksen S, Keselman HJ (1992). Backward, forward and stepwise automated subset selection algorithms: Frequency of obtaining authentic and noise variables. Br J Math Stat Psychol.

[10] Harrell FE. Regression Modeling Strategies: With Applications to Linear Models, Logistic Regression, and Survival Analysis. New York: Springer; 2001

[11] Viallefont V, Raftery AE, Richardson S (2001). Variable selection and Bayesian model averaging in case–control studies. Stat Med.

[12] Whittingham MJ, Stephens PA, Bradbury RB, Freckleton RP (2006). Why do we still use stepwise modelling in ecology and behaviour?. J Anim Ecol.

[13] Flack VF, Chang PC (1987). Frequency of Selecting Noise Variables in Subset Regression Analysis: A Simulation Study. Am Stat.

[14] Hurvich CM, Tsai CL (1990). The Impact of Model Selection on Inference in Linear Regression. Am Stat.

[15] Mundry R, Nunn Charles L (2009). Stepwise Model Fitting and Statistical Inference: Turning Noise into Signal Pollution. Am Nat.

[16] Wiegand RE (2010). Performance of using multiple stepwise algorithms for variable selection. Stat Med.

[17] Greenland S (2007). Bayesian perspectives for epidemiological research. II. Regression analysis. Int J Epidemiol.

[18] Hutmacher MM, Kowalski KG (2014). Covariate Selection in Pharmacometric Analyses: A Review of Methods. Br J Clin Pharmacol.

[19] Kadane JB, Lazar NA (2004). Methods and Criteria for Model Selection. J Am Stat Assoc.

[20] Hoeting JA, Madigan D, Raftery AE, Volinsky CT (1999). Bayesian Model Averaging: A Tutorial. Stat Sci.

[21] Tibshirani R (1996). Regression Shrinkage and Selection via the Lasso. J R Stat Soc Ser B Methodol.

[22] Zou H, Hastie T (2005). Regularization and variable selection via the elastic net. J R Stat Soc Ser B (Stat Methodol.).

[23] Heckatorn D (1997). Respondent-driven sampling: A new approach to the study of hidden population. Soc Probl.

[24] Goel S, Salganik MJ (2010). Assessing respondent-driven sampling. Proc Natl Acad Sci U S A.

[25] McCreesh N, Frost SD, Seeley J, Katongole J, Tarsh MN, Ndunguse R (2012). Evaluation of respondent-driven sampling. Epidemiology.

[26] Group EQ (1990). EuroQol - a new facility for the measurement of health-related quality of life. Health Policy.

[27] Ewing JA (1984). Detecting alcoholism. The CAGE questionnaire. JAMA.

[28] Holmes WC (1998). A short, psychiatric, case-finding measure for HIV seropositive outpatients: performance characteristics of the 5-item mental health subscale of the SF-20 in a male, seropositive sample. Med Care.

[29] Miller LC, Berg JH, Archer RL (1983). Openers - Individuals Who Elicit Intimate Self-Disclosure. J Pers Soc Psychol.

[30] Kalichman SC, Simbayi LC, Cloete A, Mthembu PP, Mkhonta RN, Ginindza T (2009). Measuring AIDS stigmas in people living with HIV/AIDS: the Internalized AIDS-Related Stigma Scale. AIDS Care.

[31] Pinel EC (1999). Stigma consciousness: the psychological legacy of social stereotypes. J Pers Soc Psychol.

[32] Venables WN, Ripley BD (2002). Modern Applied Statistics with S.

[33] Friedman J, Hastie T, Tibshirani R (2010). Regularization Paths for Generalized Linear Models via Coordinate Descent. J Stat Softw.

[34] Yang Y, Zou H (2013). An Efficient Algorithm for Computing the HHSVM and Its Generalizations. J Comput Graph Stat.

[35] Feldkircher M, Zeugner S (2009). Benchmark Priors Revisited. On Adaptive Shrinkage and the Supermodel Effect in Bayesian Model Averaging. IMF Working Papers.

[36] Hastie T, Tibshirani R, Friedman JH. The Elements of Statistical Learning: Data Mining, Inference, and Prediction. Springer; 2001

[37] Efron B (1979). Bootstrap Methods: Another Look at the Jackknife. Ann Stat.

[38] Akaike H (1974). New Look at Statistical-Model Identification. Ieee T Automat Contr.

[39] Schwarz G (1978). Estimating Dimension of a Model. Ann Stat.

[40] Sauerbrei W, Boulesteix AL, Binder H (2011). Stability investigations of multivariable regression models derived from low- and high-dimensional data. J Biopharm Stat.

[41] Zou H (2006). The Adaptive Lasso and Its Oracle Properties. J Am Stat Assoc.

[42] Zou H, Zhang HH (2009). On the Adaptive Elastic-Net with a Diverging Number of Parameters. Ann Stat.

[43] Browne MW (2000). Cross-Validation Methods. J Math Psychol.

[44] Gelman A, Carlin JB, Stern HS, Rubin DB (2003). Bayesian Data Analysis.

[45] Raftery AE, Madigan D, Hoeting JA (1997). Bayesian Model Averaging for Linear Regression Models. J Am Stat Assoc.

[46] Wasserman L (2000). Bayesian Model Selection and Model Averaging. J Math Psychol.

[47] Barbieri MM, Berger JO (2004). Optimal predictive model selection. Ann Stat.

[48] Genell A, Nemes S, Steineck G, Dickman PW (2010). Model selection in medical research: a simulation study comparing Bayesian model averaging and stepwise regression. BMC Med Res Methodol.

[49] Tibshirani R (1997). The lasso method for variable selection in the Cox model. Stat Med.

[50] Ribbing J, Nyberg J, Caster O, Jonsson EN (2007). The lasso--a novel method for predictive covariate model building in nonlinear mixed effects models. J Pharmacokinet Pharmacodyn.

[51] Sauerbrei W, Royston P, Binder H (2007). Selection of important variables and determination of functional form for continuous predictors in multivariable model building. Stat Med.

[52] Rentsch C, Bebu I, Guest JL, Rimland D, Agan BK, Marconi V (2014). Combining epidemiologic and biostatistical tools to enhance variable selection in HIV cohort analyses. PLoS One.

[53] Burnham KP, Anderson DR, Huyvaert KP (2011). AIC model selection and multimodel inference in behavioral ecology: some background, observations, and comparisons. Behav Ecol Sociobiol.

[54] van de Geer SA, Buhlmann P. On the conditions used to prove oracle results for the Lasso. Elec J of Stat. 2009;3:1360-1392.

[55] Glmnet Vignette. [http://web.stanford.edu/~hastie/glmnet/glmnet_alpha.html] Accessed: September 19, 2014.

[56] Montgomery JM, Nyhan B (2010). Bayesian Model Averaging: Theoretical Developments and Practical Applications. Polit Anal.

[57] Dietze P, Stoove M, Miller P, Kinner S, Bruno R, Alati R (2010). The self-reported personal wellbeing of a sample of Australian injecting drug users. Addiction.

[58] Douab T, Marcellin F, Vilotitch A, Protopopescu C, Preau M, Suzan-Monti M (2014). Health-related quality of life of people living with HIV followed up in hospitals in France: comparing trends and correlates between 2003 and 2011 (ANRS-VESPA and VESPA2 national surveys). AIDS Care.

[59] Jelsma J, Maclean E, Hughes J, Tinise X, Darder M (2005). An investigation into the health-related quality of life of individuals living with HIV who are receiving HAART. AIDS Care.

[60] Preau M, Protopopescu C, Spire B, Sobel A, Dellamonica P, Moatti JP (2007). Health related quality of life among both current and former injection drug users who are HIV-infected. Drug Alcohol Depend.

