# Supplementary material for: Comparison of subset selection methods in linear regression in the context of health-related quality of life and substance abuse in Russia
Source: BMC Med Res Methodol. 2015 Aug 30;15:71. doi: 10.1186/s12874-015-0066-2 (PMC4553217; doi:10.1186/s12874-015-0066-2)
Supplement: Additional file 1: — Study questionnaire (selected questions). Study questions used to create variables for the analysis. (PDF 187 kb) [file 12874_2015_66_MOESM1_ESM.pdf]

Additional file 1: Study questionnaire (selected questions)

QA04 Date of interview ………………… ………………… ………………..

Day *Month* *Year*

SECTION B: DEMOGRAPHIC AND SOCIOECONOMIC CHARACTERISTICS

QB01 When were you born?

__ __ __ __ __ __

Month *Year*

QB02 Sex

1. Male
2. Female

QB07 What is your formal education (highest education certificate)?

1. No formal education/never attended school
2. Primary education
3. Basic education (through 9th grade)
4. Secondary education (through 11th grade)
5. Vocational education
6. Any higher education
7. Other (please specify ……………………………………………………)

QB08 During the last **6 months** what was your source **(QB08**) and main source **(QB08A)** of money for you to live on....

*[Do not read out response options***.** *NB! QB08 possible more than one response, QB08A circle one response.]*

		QB08 Source	QB08A Main source
1.	Regular official job, employed with a regular salary (full or part-time)	1	1
2.	Regular unofficial job, employed with a regular salary (full or part-time)	1	1
3.	Temporary work (include odd jobs, off-the--books, etc.)	1	1
4.	Work at family business or farm	1	1
5.	Self-employed (in a particular trade)	1	1
6.	Government benefits (social welfare, unemployment insurance, sick leave etc.)	1	1
7.	Spouse, partner, relative, or friend's income	1	1

8.	Student financial aid/loans/grants	1	1
9.	Street begging/panhandling etc	1	1
10.	Selling drugs	1	1
11.	Sex for money	1	1
12.	Theft, robbing, or stealing	1	1
13.	Other (please specify ………………………………………………………………………)	1	1
99.	No answer	1	1

QB12 During the last **6 months**, where did you live most of the time?
[Do not read out response options. Circle one response.]

1. My own (or my spouse or partner's) house, flat, or apartment (owned not rented)
2. House, flat, apartment, or room rented (leased) by me (or my spouse or partner)
2a. Communal apartment, owned or rented
3. Dormitory, hostel (room rented for daily basis)
4. Someone else's (including parents, relatives, friends) house flat or apartment
5. Shelter, welfare residence
6. No fixed address (e.g., street, park, abandoned building)
7. Residential community
8. Drug treatment institution
9. Other treatment institution/hospital
10. Jail/prison
11. Other (please specify…………………………….............………………….)

QB11 What is your **current** marital status?
[Do not read out response options. Circle one response.]

1. Legally married
2. Living as married ("common law")
3. Widowed
4. Divorced
5. Never married/single

QB13B Do you have basic medical insurance?
0. No
1. Yes
88. Don't know

QB14 How would you describe coping with your income today?
[Read out each category in turn. Circle one response.]

1.Living comfortably on present income
2. Coping on present income
3. Finding it difficult to cope on present income
4. Finding it very difficult to cope on present income
88. Don't know

SECTION C: CONTACT WITH TREATMENT SERVICES AND PRISON

QC01 Have you **ever** received any treatment intended to help you modify your drug use?

0. No
1. Yes

The next questions are for the respondents who ever received some narcological treatment
QC01C Have you ever had difficulty obtaining narcological treatment?

0. No
1. Yes
88. Don't know

QC04 Have you **ever** been in jail/prison?

0. No
1. Yes

SECTION D: ALCOHOL AND DRUG USE

QD09 Questions about your alcohol use.
[Read out each category in turn, mark the correct number for each.]

		No	Yes
1.	Have you **ever** felt you should cut down on your drinking?	0	1
2.	Have people annoyed you by criticizing your drinking?	0	1
3.	Have you **ever** felt bad or guilty about your drinking?	0	1
4.	Have you **ever** had a drink first thing in the morning to steady your nerves or get rid of a hangover (eye-opener)?	0	1

QD10 Had you **ever** used some illegal drug in some other way (pills, snorting or smoking) before you started injected yourself?

0. No
1. Yes

QD11A How old were you when you **first used** any drug any means of administration other than injection (opiates, cocaine, amphetamines, other stimulants, barbiturates, tranquilizers, sedatives, thinners, etc)?
[*NB! Only document narcotic and/or psychotropic drugs not prescribed by a doctor]*

…………………………years old
88. Don't know

QD11A1 Did you **use** a drug other than cannabis by any means of administration other than injection (opiates, cocaine, amphetamines, other stimulants, barbiturates, tranquilizers, sedatives, thinners, etc)?
[*NB! Only document narcotic and/or psychotropic drugs not prescribed by a doctor]*

QD11 How old were you when you **first used** a drug other than cannabis by any means of administration other than injection (opiates, cocaine, amphetamines, other stimulants, barbiturates, tranquilizers, sedatives, thinners, etc)?
[*NB! Only document narcotic and/or psychotropic drugs not prescribed by a doctor]*

………………………years old
88. Don't know

QD13 How old were you when you **first** injected a drug for non-medical purpose?

………………………years old
88. Don't Know

QD17 In how many days of the **4 weeks** did you inject drugs?

……………………… number of days
88. Don't know

QD16 On the **last day** you injected, how many times did you inject?

……………………… times a day
88. Don't know

QD18 Now I am going to ask you some questions about specific drugs you have used in the last **4 weeks**. For each drug mentioned, I am going to ask separately if you have injected the drug in the last **4 weeks (QD18B)**, and which was the **main** drug you injected in the last 4 weeks **(QD18C)**:

Drug	QD18B Injection in last 4 weeks		QD18C Main injected drug last 4 weeks (tick only one)
	Yes	No	
1. Fentanyl (China White, White Persian / Afghan)	1	0	
2. Amphetamine	1	0	
3. MAK (Poppy liquid)	1	0	
4. Heroin	1	0	
5. Methampetamine	1	0	
6. Cocaine	1	0	
7. Ecstasy, MDMA	1	0	
8. Sudafed	1	0	
9A DESOMORPHINE (crocodile)			
9B Methadone			
9. GBL, GHB, ketamine	1	0	
10. Other (please specify………………)	1	0	
11. Other (please specify………………)	1	0	

SECTION E: HIV RISK PRACTICES ASSOCIATED WITH INJECTING DRUGS

QE01 In the **last 4 weeks** how many times did you inject with needles and/or syringes previously used by someone else?

……………………… (number of times)
88 Don't know

QE04 In the **last4 weeks**, did you get <u>any</u> new and unused needles and/or syringes?

0. No
1. Yes

QE13 In the last **6 months**, did the police or other authorities ever confiscate any injecting equipment from you?

0. No
1. Yes

QE14 Have you **ever** injected with a needle and/or syringe that someone else had already used?

0. No→ SECTION F
1. Yes

SECTION F: SEXUAL BEHAVIOR

QF02A During the last 6 months have you had sexual intercourses with the partners of the opposite sex, same sex as yours, both sexes (whether they were your regular partners, occasional partners or commercial)

1. Only from the opposite sex
2. Only from the same sex as mine
3. Both sexes
99. If has not had intercourse

QF02B How many partners from the opposite sex did you have **during past six months**?

	Number
1. Occasional	
2. Regular	
3. Partners, whom you gave the presents or money as an exchange for sexual intercourse	
4. Partners, who gave you the presents or money as an exchange for sexual intercourse	

QF02C How many partners from the same sex as yours did you have during past six months?

	Number
1. Occasional	
2. Regular	
3. Partners, whom you gave the presents or money as an exchange for sexual intercourse	
4. Partners, who gave you the presents or money as an exchange for sexual intercourse	

QF12 As far as you know, have any of these opposite-sex primary partners you had sex with within the **last 6 months** have **ever** ... *[Read out each item in turn. Circle one response for each.]*

		No	Yes
1.	...been told that they were HIV positive or that they had the AIDS?	0	1
2.	...been diagnosed with hepatitis?	0	1

QF06A Did you use condom at the last sexual intercourse?

0. No
1. Yes
88, Do not know

SECTION G Knowledge – HIV, tuberculosis, hepatitis

QG07. Have you ever been vaccinated against hepatitis B (HBV)?

0. No
1. Yes
88. Don't know

QG08. How many HBV vaccination shots did you get in all?

_____shots
88. Don't know

SECTION K: OVERDOSE

QK06 Have you **ever** overdosed on narcotics to the point where you lost consciousness?

0. No
1. Yes

SECTION H: PHYSICAL AND PSYCHOLOGICAL HEALTH AND USE OF TREATMENT SERVICES

QH01B Please indicate which statements best describe your own health state **today**.

QH01B1 Mobility

1. I have no problems in walking about
2. I have some problems in walking about
3. I am confined to bed

QH01B2 Self-Care

1. I have no problems with self-care
2. I have some problems washing or dressing myself
3. I am unable to wash or dress myself

QH01B3 Usual Activities (*e.g. work, study, housework, family or leisure activities)*

1. I have no problems with performing my usual activities
2. I have some problems with performing my usual activities
3. I am unable to perform my usual activities

QH01B4 Pain/Discomfort

1. I have no pain or discomfort
2. I have moderate pain or discomfort
3. I have extreme pain or discomfort

QH01B5 Anxiety/Depression

1. I am not anxious or depressed
2. I am moderately anxious or depressed
3. I am extremely anxious or depressed

QH01C To help people say how good or bad a health state is, we have drawn a scale (rather like a thermometer) on which the best state you can imagine is marked 100 and the worst state you can imagine is marked 0. We would like you to indicate on this scale how good or bad your own health is today, in your opinion. Please do this by drawing a line from the box below to whichever point on the scale indicates how good or bad your health state is **today**.
[PROMPT CARD D]

.... (number)

QH06 What type of health care services have you received **in last 12 months**?
[Do not read out response options. Please circle all that are applicable]

1. I have not received any health care in last 12 months
2. Family doctor/general practitioner
3. Dentist
4. Infectious diseases doctor
5. Psychiatrist
6. Drug abuse treatment/substitution treatment
7. Emergency department/ambulance
8. Lung specialist (pulmonologist)
10. Psycologist
11. Physical therapist
12. TB doctor
13, Surgeon
9. Other (please specify …………………………………………………….)

QH08C Have you ever had difficulty obtaining medical care because of your drug use?

0. No
1. Yes
88. Don't know

HIV-TESTING AND HIV TREATMENT

QH11 Have you **ever** been tested for the HIV?

0. No
1. Yes
88. Don't know

QH11A Have you ever been told that you are HIV infected?

0. No
1. Yes
88. Don't know

QH14 What was the result of your **last** HIV test?

0. Negative (not infected)
1. Positive (infected)
2. Inconclusive
3. Did not get the result
88. Don't know
99. No answer

The following are questions only for those, who are HIV-infected

QH16 Do you receive regular HIV care? By regular HIV care we mean visiting HIV physician at least once in a six months.

0. No
1. Yes
88. Don't know

TUBERCULOSIS

QH38 Have you **ever** been told by a doctor that you had tuberculosis?

0. No
1. Yes
88. Don't know

HEPATITIS

QH41 Has any doctor/medical personnel **ever** told you that you have hepatitis B?

0. No
1. Yes

QH44 Has any doctor/medical personnel **ever** told you that you have hepatitis C?

0. No
1. Yes

QH46 Have you **ever** been offered antiviral therapy for HCV?

0. No
1. Yes

QH46A Have you **ever** received antiviral therapy for HCV?

0. No
1. Yes →QH46C

MENTAL HEALTH

QH47 How much of the time during the last **four weeks** have you…?

Response options:

1 - all of the time; **2**- most of the time; **3** - a good bit of the time; 4 - some of the time; **5** - a little of the time; **6** - or none of the time.

		1	2	3	4	5	6
1.	…been a very nervous person?						
2.	…felt downhearted and depressed?						
3.	…felt calm and peaceful?						
4.	…felt so down in the dumps that nothing could cheer you up?						
5.	..been a happy person?						

SECTION I: USE OF TREATMENT, HIV PREVENTION, SOCIAL WELFARE AND HARM REDUCTION SERVICES

QI01 Have you needed the following services within last **6 months (QI02)** and have you received the following services within last **6 months (QI02)**?

			**QI01: Needed services**		**QI02: Received services**		
		Do not know that the service exists	No	Yes	No	Yes	Don’t know
1.	Syringe exchange services	0	1	2	1	2	88
2.	Drug detoxification	0	1	2	1	2	88
3.	HIV testing	0	1	2	1	2	88
4.	HIV-treatment and care	0	1	2	1	2	88
5.	Mental health services	0	1	2	1	2	88
6.	HIV testing of sexual partner	0	1	2	1	2	88
7.	Social counseling	0	1	2	1	2	88
8.	Social housing	0	1	2	1	2	88
9.	Child care	0	1	2	1	2	88
10	Psychological counseling	0	1	2	1	2	88
11	TB testing/treatment	0	1	2	1	2	88
12	Hepatitis C testing/treatment	0	1	2	1	2	88
13	Legal Advice	0	1	2	1	2	88
14	Migrant support service	0	1	2	1	2	88
15	Employment support	0	1	2	1	2	88
16	Other	0	1	2	1	2	88

SECTION J: HIV AND IDU STATUS DISCLOSURE AND STIGMA

QJ01 Questions about disclosure of IDU status.

NB! The following questions are scored on a scale of 1-5 (1 - Have not discussed at all; 5 - Have discussed fully and completely)

1.	To what extent have you discussed your status as a drug user with your main sexual partner?	1	2	3	4	5
2.	To what extent have you discussed your status as a drug user with casual sexual partners?	1	2	3	4	5
3.	To what extent have you discussed your status as a drug user with close friends?	1	2	3	4	5
4.	To what extent have you discussed your status as a drug user with your parents?	1	2	3	4	5
5.	To what extent have you discussed your status as a drug user with other family members?	1	2	3	4	5
6.	To what extent have you discussed your status as a drug user with your employer or potential employers?	1	2	3	4	5
7.	To what extent have you discussed your status as a drug user with your health care providers?	1	2	3	4	5

QJ02 Statements on the internalization and consciousness of IDU stigma.

NB! The following statements are scored on a scale of 1-5 (1 - Strongly Agree; 2 - Agree; 3 - Undecided ; 4- Disagree;5 - Strongly Disagree)

1.	It is difficult to tell people about being a drug user.	1	2	3	4	5
2.	Being a drug user makes me feel dirty	1	2	3	4	5
3.	I feel guilty about using drugs.	1	2	3	4	5
4.	I am ashamed that use drugs.	1	2	3	4	5
5.	I sometimes feel worthless because I am a drug user.	1	2	3	4	5
6.	I hide my status as a drug user from others.	1	2	3	4	5
7.	Stereotypes about drug users have not affected me personally.	1	2	3	4	5
8.	Most people do not pass judgments on drug users.	1	2	3	4	5
9.	My being a drug user does not influence how others interact with me.	1	2	3	4	5
10.	I almost never think about the fact that I am a drug user when I interact with others.	1	2	3	4	5
11.	Most people have a lot more negative thoughts about drug users than they actually express.	1	2	3	4	5
12.	Most people have a problem viewing drug users as equals.	1	2	3	4	5
