# Supplementary material for: Comparison of subset selection methods in linear regression in the context of health-related quality of life and substance abuse in Russia
Source: BMC Med Res Methodol. 2015 Aug 30;15:71. doi: 10.1186/s12874-015-0066-2 (PMC4553217; doi:10.1186/s12874-015-0066-2)
Supplement: Additional file 2: — Variable codes. Provides detailed description of variable codes used in the main text and other additional files. (PDF 60 kb) [file 12874_2015_66_MOESM2_ESM.pdf]

Additional file 2: Variable codes

For simplicity we are using variable codes instead of full variable names throughout all Additional files.
The following table provides reference for the codes used ("-" denotes the reference category):

Variable names	Variable codes	Variable names	Variable codes
**Sex**:<br>Male<br>Female	-<br>sex	**Paid for sex in the last 6 months**:<br>No<br>Yes	-<br>pay.sex.6m
**Age (median = 32 y.o)**:<br>Less than 32 y.o.<br>32 y.o. or older	-<br>age.d	**HIV and Hepatitis C status of primary sexual partner**:<br>HIV and HCV negative or unknown<br>Known to be HIV or HCV positive<br>No primary partner in the last 6 months	-<br>HIV.HC.partner_1<br>HIV.HC.partner_2
**Education**:<br>Primary or Basic<br>Secondary, Vocational or at least some Higher	-<br>education	**Ever been tested for HIV**:<br>No (or don't know)<br>Yes	-<br>HIV.test
**Main source of income**:<br>Legal source<br>Illegal source	-<br>income.source	**HIV status (based on study testing)**:<br>Negative<br>Positive	-<br>HIV.status
**Level of income**:<br>Coping well<br>Coping is difficult (or very difficult)	-<br>income.level	**Receiving regular HIV care**:<br>HIV-negative or unaware<br>HIV+; receives regular HIV care<br>HIV+; does not receive regular HIV care	-<br>HIV.care_1<br>HIV.care_2
**Living arrangements**:<br>Someone else's house<br>Owned or rented place<br>Shelter / no fixed place	-<br>living.sit_1<br>living.sit_2	**Tuberculosis history awareness**:<br>No (or don't know)<br>Yes	-<br>TB
**Marital status**:<br>Not married<br>Married	-<br>marital	**Treatment of Hepatitis C**:<br>Never diagnosed with HCV<br>HCV+, never been offered treatment<br>HCV+, was offered treatment, but did not receive it<br>HCV+, was offered treatment and received it	-<br>HepC.treatment_1<br>HepC.treatment_2<br>HepC.treatment_3
**Alcohol abuse using CAGE scale**:<br>CAGE = 0-1<br>CAGE = 2-4	-<br>CAGE	**Hepatitis B history awareness**:<br>No<br>Yes	-<br>HepB.aware
**Age of first drug use (cannabis excluded; median = 16 y.o.)**:<br>17 y.o. or older<br>16 y.o. or younger	-<br>age.drug.use	**Ever vaccinated against Hepatitis B**:<br>No (or don't know)<br>Yes (at least one dose)	-<br>HepB.vaccine

Variable names	Variable codes	Variable names	Variable codes
**Main drug of use**:<br>(Meth)-amphetamines<br>Methadone / Fentanyl<br>Heroin	-<br>main.drug_1<br>main.drug_2	**History of incarceration**:<br>No<br>Yes	-<br>incarceration
**Poly-drug use in the last 4 weeks**:<br>Injected 1 class of drugs<br>Injected 2 or more classes of drugs	-<br>poly.drug	**Having basic medical insurance**:<br>No<br>Yes	-<br>med.insurance
**Frequency of injecting drugs (days during the last 4 weeks; median=20)**:<br>19 days or less<br>20 days or more	-<br>drug.freq.days	**Receiving any healthcare services in the last 12 months**:<br>Received<br>Not received	-<br>med.care.12m
**Frequency of injecting drugs (times per day; median = 1)**:<br>One<br>Two or more	-<br>drug.freq.times	**Receiving detoxification services in the last 6 months**:<br>Did not need detox services<br>Needed, but did not receive detox<br>Needed and received detox	-<br>detox_1<br>detox_2
**Used non-sterile injecting equipment at least once in the last 4 weeks**:<br>No (or don't know)<br>Yes	-<br>inject.used.recent	**Ever had difficulties obtaining drug abuse treatment**:<br>Never received treatment (or don't know)<br>Had no difficulties<br>Had difficulties	-<br>drug.treat.problems_1<br>drug.treat.problems_2
**Ever used non-sterile injecting equipment**:<br>No<br>Yes	-<br>Inject.used.ever	**Had difficulties obtaining medical care because of drug use**:<br>No (or don't know)<br>Yes	-<br>med.care.problems
**Getting sterile injecting equipment (in last 4 weeks)**:<br>No<br>Yes	-<br>get.unused.syr	**Ever experienced police confiscate syringes**:<br>No<br>Yes	-<br>police.confiscate.syr
**Ever overdosed**:<br>No<br>Yes	-<br>overdose	**PWID status disclosure to family or friends**:<br>Rather disclosed<br>Rather did not disclose	IDU.disclosure.close
**Mental health problems score**:<br>Lower score on mental health problems<br>Higher score on mental health problems	-<br>MHI5	**PWID status disclosure to a healthcare provider**:<br>Rather disclosed<br>Rather did not disclose	-<br>IDU.disclosure.doctor
**Sexually active in the last 6 months**:<br>No<br>Yes	-<br>sex.active	**Internalized PWID stigma**:<br>Low<br>High	-<br>IDU.stigma.internal
**Involved in sexual work in the last 6 months**:<br>No<br>Yes	-<br>sell.sex.6m	**PWID stigma consciousness**:<br>Low<br>High	-<br>IDU.stigma.conscious
