# Supplementary material for: Comparison of subset selection methods in linear regression in the context of health-related quality of life and substance abuse in Russia
Source: BMC Med Res Methodol. 2015 Aug 30;15:71. doi: 10.1186/s12874-015-0066-2 (PMC4553217; doi:10.1186/s12874-015-0066-2)
Supplement: Additional file 3: — Stepwise regression. The regression coefficients along with their 95 % CIs, and the bootstrap inclusion frequencies of infependent variables for the models selected using automatic stepwise selection algorithm (backward elimination and forward selection) with AIC, BIC and the Likelihood ratio test (p = 0.05). (PDF 229 kb) [file 12874_2015_66_MOESM3_ESM.pdf]

Additional file 3: Stepwise regression

Note: Inclusion frequency is estimated using bootstrap (number of iterations = 2,000).

Table S3.1. Backward elimination regression using AIC

Variables	Backward elimination, AIC			
	Beta	95% CI (asymptotic)	95% CI (bootstrap)	Inclusion frequency
(Intercept)	79.57	(73.96 ; 85.18)	(73.31 ; 85.83)	1.00
sex	-2.39	(-4.58 ; -0.2)	(-4.41 ; -0.37)	0.86
age.d	-4.76	(-6.57 ; -2.95)	(-6.47 ; -3.05)	1.00
education	3.18	(-0.1 ; 6.47)	(-0.66 ; 7.03)	0.72
income.source				0.16
income.level	-3.76	(-5.77 ; -1.74)	(-5.86 ; -1.65)	0.99
living.sit_1	1.42	(-0.51 ; 3.36)	(-0.49 ; 3.34)	0.59
living.sit_2				0.20
marital				0.31
CAGE	-1.80	(-3.85 ; 0.25)	(-3.88 ; 0.28)	0.62
age.drug.use	-3.40	(-5.36 ; -1.45)	(-5.36 ; -1.44)	0.96
main.drug_1				0.29
main.drug_2				0.22
poly.drug	-3.61	(-6.24 ; -0.99)	(-6.14 ; -1.09)	0.90
drug.freq.days	-1.78	(-3.76 ; 0.2)	(-3.92 ; 0.36)	0.50
drug.freq.times				0.24
inject.used.recent	-2.16	(-4.23 ; -0.09)	(-4.53 ; 0.21)	0.71
inject.used.ever				0.24
get.unused.syr				0.23
overdose				0.20
MHI5	-2.62	(-4.58 ; -0.65)	(-4.71 ; -0.52)	0.85
sex.active				0.36
sell.sex.6m				0.44
pay.sex.6m	2.70	(-0.68 ; 6.08)	(-0.25 ; 5.65)	0.46
HIV.HC.partner_1	-2.08	(-4.86 ; 0.7)	(-5.13 ; 0.98)	0.53
HIV.HC.partner_2	-2.20	(-4.68 ; 0.29)	(-4.88 ; 0.49)	0.53
HIV.test	3.46	(-0.28 ; 7.2)	(-0.48 ; 7.41)	0.66
HIV.status	-1.88	(-4.16 ; 0.39)	(-4.2 ; 0.43)	0.63
HIV.care_1				0.37
HIV.care_2	-5.32	(-7.89 ; -2.75)	(-8.03 ; -2.61)	0.86
TB	-3.39	(-6.91 ; 0.13)	(-6.5 ; -0.27)	0.72
HepC.treatment_1	-3.96	(-6.29 ; -1.63)	(-6.38 ; -1.54)	0.96
HepC.treatment_2	-6.42	(-10.39 ; -2.44)	(-11.47 ; -1.37)	0.93
HepC.treatment_3				0.32
HepB.aware				0.22
HepB.vaccine	1.79	(-0.29 ; 3.88)	(-0.33 ; 3.91)	0.60
incarceration	-1.52	(-3.44 ; 0.41)	(-3.36 ; 0.33)	0.54
med.insurance				0.48
med.care.12m	-1.52	(-3.45 ; 0.4)	(-3.44 ; 0.39)	0.51
detox_1	-5.57	(-8.55 ; -2.58)	(-8.67 ; -2.46)	0.96
detox_2	-4.78	(-7.99 ; -1.57)	(-7.75 ; -1.81)	0.94
drug.treat.problems_1	1.56	(-0.33 ; 3.44)	(-0.37 ; 3.48)	0.48
drug.treat.problems_2				0.30
med.care.problems	-4.35	(-8.12 ; -0.58)	(-8.02 ; -0.69)	0.79
police.confiscate.syr				0.24
IDU.disclosure.close				0.25
IDU.disclosure.doctor				0.23
IDU.stigma.internal	-3.94	(-5.97 ; -1.91)	(-6.02 ; -1.86)	0.98
IDU.stigma.conscious	1.42	(-0.46 ; 3.31)	(-0.56 ; 3.41)	0.56
**Adjusted R^2**	**0.38**			

Table S3.2. Backward elimination regression using BIC

Variables	Backward elimination, BIC			
	Beta	95% CI (asymptotic)	95% CI (bootstrap)	Inclusion frequency
(Intercept)	82.69	(79.84 ; 85.55)	(79.99 ; 85.4)	1.00
sex				0.31
age.d	-4.49	(-6.24 ; -2.75)	(-6.2 ; -2.79)	0.99
education				0.39
income.source				0.02
income.level	-4.78	(-6.76 ; -2.81)	(-6.81 ; -2.76)	0.96
living.sit_1				0.13
living.sit_2				0.03
marital				0.03
CAGE				0.33
age.drug.use	-3.36	(-5.26 ; -1.46)	(-5.25 ; -1.48)	0.76
main.drug_1				0.08
main.drug_2				0.05
poly.drug	-3.70	(-6.29 ; -1.11)	(-6.12 ; -1.28)	0.59
drug.freq.days				0.31
drug.freq.times				0.10
inject.used.recent	-2.61	(-4.6 ; -0.62)	(-4.78 ; -0.44)	0.48
inject.used.ever				0.05
get.unused.syr				0.03
overdose				0.03
MHI5	-3.50	(-5.4 ; -1.6)	(-5.56 ; -1.45)	0.61
sex.active				0.13
sell.sex.6m				0.06
pay.sex.6m				0.14
HIV.HC.partner_1				0.21
HIV.HC.partner_2				0.20
HIV.test				0.28
HIV.status				0.34
HIV.care_1				0.21
HIV.care_2	-7.17	(-9.25 ; -5.09)	(-9.43 ; -4.92)	0.80
TB				0.30
HepC.treatment_1	-4.21	(-6.48 ; -1.95)	(-6.62 ; -1.8)	0.77
HepC.treatment_2	-6.08	(-10.07 ; -2.09)	(-10.89 ; -1.27)	0.63
HepC.treatment_3				0.04
HepB.aware				0.03
HepB.vaccine	2.89	(0.9 ; 4.88)	(0.85 ; 4.93)	0.33
incarceration				0.17
med.insurance				0.18
med.care.12m				0.15
detox_1	-4.41	(-7.28 ; -1.54)	(-7.47 ; -1.34)	0.77
detox_2				0.56
drug.treat.problems_1				0.16
drug.treat.problems_2				0.15
med.care.problems	-5.44	(-9.2 ; -1.69)	(-9.29 ; -1.6)	0.46
police.confiscate.syr				0.04
IDU.disclosure.close				0.07
IDU.disclosure.doctor				0.04
IDU.stigma.internal	-3.36	(-5.34 ; -1.38)	(-5.41 ; -1.3)	0.88
IDU.stigma.conscious				0.14
**Adjusted R^2**	**0.35**			

Table S3.3. Backward elimination regression using Likelihood Ratio Test (p=0.05)

Variables	Backward elimination, LRT (p=0.05)			
	Beta	95% CI (asymptotic)	95% CI (bootstrap)	Inclusion frequency
(Intercept)	77.57	(72.44 ; 82.71)	(71.71 ; 83.43)	1.00
sex	-2.43	(-4.59 ; -0.27)	(-4.41 ; -0.44)	0.66
age.d	-5.00	(-6.78 ; -3.22)	(-6.73 ; -3.27)	1.00
education	3.49	(0.22 ; 6.77)	(-0.42 ; 7.4)	0.56
income.source				0.06
income.level	-4.58	(-6.54 ; -2.62)	(-6.58 ; -2.59)	0.98
living.sit_1				0.35
living.sit_2				0.08
marital				0.13
CAGE				0.47
age.drug.use	-3.53	(-5.43 ; -1.62)	(-5.42 ; -1.64)	0.90
main.drug_1				0.14
main.drug_2				0.10
poly.drug	-3.51	(-6.09 ; -0.92)	(-5.96 ; -1.05)	0.79
drug.freq.days				0.39
drug.freq.times				0.14
inject.used.recent	-2.84	(-4.82 ; -0.87)	(-5 ; -0.69)	0.59
inject.used.ever				0.10
get.unused.syr				0.09
overdose				0.09
MHI5	-3.09	(-4.98 ; -1.2)	(-5.12 ; -1.06)	0.75
sex.active				0.24
sell.sex.6m				0.22
pay.sex.6m				0.29
HIV.HC.partner_1				0.34
HIV.HC.partner_2				0.34
HIV.test	3.96	(0.3 ; 7.62)	(0.15 ; 7.77)	0.49
HIV.status				0.48
HIV.care_1				0.26
HIV.care_2	-7.32	(-9.42 ; -5.22)	(-9.58 ; -5.06)	0.81
TB	-4.06	(-7.56 ; -0.55)	(-7.17 ; -0.94)	0.53
HepC.treatment_1	-4.39	(-6.66 ; -2.11)	(-6.79 ; -1.99)	0.90
HepC.treatment_2	-6.61	(-10.58 ; -2.64)	(-11.44 ; -1.78)	0.83
HepC.treatment_3				0.14
HepB.aware				0.08
HepB.vaccine	2.34	(0.33 ; 4.36)	(0.29 ; 4.4)	0.46
incarceration				0.34
med.insurance				0.31
med.care.12m				0.33
detox_1	-5.52	(-8.4 ; -2.64)	(-8.55 ; -2.48)	0.91
detox_2	-4.06	(-7.23 ; -0.89)	(-6.97 ; -1.14)	0.81
drug.treat.problems_1				0.31
drug.treat.problems_2				0.21
med.care.problems	-5.28	(-9.01 ; -1.54)	(-8.97 ; -1.58)	0.64
police.confiscate.syr				0.10
IDU.disclosure.close				0.13
IDU.disclosure.doctor				0.10
IDU.stigma.internal	-3.39	(-5.37 ; -1.42)	(-5.41 ; -1.38)	0.94
IDU.stigma.conscious				0.33
**Adjusted R^2**	**0.37**			

Table S3.4. Forward selection regression using AIC

Variables	Forward selection, AIC			
	Beta	95% CI (asymptotic)	95% CI (bootstrap)	Inclusion frequency
(Intercept)	75.89	(70.15 ; 81.64)	(69.19 ; 82.6)	1.00
sex	-2.59	(-4.76 ; -0.42)	(-4.58 ; -0.6)	0.81
age.d	-4.82	(-6.64 ; -3.01)	(-6.55 ; -3.1)	1.00
education	2.97	(-0.31 ; 6.25)	(-0.83 ; 6.76)	0.70
income.source				0.15
income.level	-3.88	(-5.88 ; -1.89)	(-5.91 ; -1.85)	0.99
living.sit_1	1.49	(-0.44 ; 3.41)	(-0.41 ; 3.39)	0.56
living.sit_2				0.20
marital				0.23
CAGE	-1.68	(-3.71 ; 0.36)	(-3.75 ; 0.4)	0.60
age.drug.use	-3.37	(-5.33 ; -1.41)	(-5.32 ; -1.42)	0.95
main.drug_1				0.16
main.drug_2				0.18
poly.drug	-3.66	(-6.29 ; -1.03)	(-6.22 ; -1.1)	0.90
drug.freq.days	-1.91	(-3.88 ; 0.07)	(-4.05 ; 0.23)	0.55
drug.freq.times				0.24
inject.used.recent	-2.47	(-4.52 ; -0.42)	(-4.81 ; -0.12)	0.73
inject.used.ever				0.23
get.unused.syr				0.20
overdose				0.19
MHI5	-2.76	(-4.71 ; -0.82)	(-4.84 ; -0.68)	0.86
sex.active	1.50	(-0.58 ; 3.58)	(-0.64 ; 3.64)	0.43
sell.sex.6m				0.35
pay.sex.6m				0.41
HIV.HC.partner_1				0.37
HIV.HC.partner_2				0.32
HIV.test	3.48	(-0.23 ; 7.2)	(-0.39 ; 7.36)	0.65
HIV.status	-1.93	(-4.17 ; 0.32)	(-4.16 ; 0.31)	0.55
HIV.care_1				0.26
HIV.care_2	-5.60	(-8.16 ; -3.05)	(-8.31 ; -2.89)	1.00
TB	-3.53	(-7.05 ; -0.02)	(-6.7 ; -0.36)	0.72
HepC.treatment_1	-4.20	(-6.51 ; -1.89)	(-6.56 ; -1.83)	0.92
HepC.treatment_2	-6.33	(-10.31 ; -2.36)	(-11.28 ; -1.39)	0.89
HepC.treatment_3				0.34
HepB.aware				0.24
HepB.vaccine	2.07	(0 ; 4.14)	(-0.02 ; 4.16)	0.62
incarceration	-1.45	(-3.38 ; 0.48)	(-3.3 ; 0.4)	0.50
med.insurance	1.61	(-0.62 ; 3.84)	(-0.78 ; 4.01)	0.49
med.care.12m				0.50
detox_1	-6.05	(-9.01 ; -3.08)	(-9.17 ; -2.93)	0.96
detox_2	-4.28	(-7.47 ; -1.1)	(-7.19 ; -1.38)	0.93
drug.treat.problems_1	1.49	(-0.4 ; 3.37)	(-0.44 ; 3.42)	0.43
drug.treat.problems_2				0.35
med.care.problems	-4.34	(-8.11 ; -0.58)	(-8.02 ; -0.67)	0.79
police.confiscate.syr				0.22
IDU.disclosure.close				0.23
IDU.disclosure.doctor				0.20
IDU.stigma.internal	-4.17	(-6.19 ; -2.15)	(-6.24 ; -2.1)	0.98
IDU.stigma.conscious	1.54	(-0.35 ; 3.43)	(-0.46 ; 3.55)	0.53
**Adjusted R^2**	**0.38**			

Table S3.5. Forward selection regression using BIC

Variables	Forward selection, BIC			
	Beta	95% CI (asymptotic)	95% CI (bootstrap)	Inclusion frequency
(Intercept)	82.52	(79.65 ; 85.39)	(79.82 ; 85.22)	1.00
sex				0.20
age.d	-4.37	(-6.11 ; -2.63)	(-6.11 ; -2.64)	0.99
education				0.36
income.source				0.02
income.level	-4.62	(-6.61 ; -2.63)	(-6.64 ; -2.6)	0.97
living.sit_1				0.10
living.sit_2				0.04
marital				0.01
CAGE				0.37
age.drug.use	-3.42	(-5.33 ; -1.5)	(-5.3 ; -1.54)	0.63
main.drug_1				0.02
main.drug_2				0.07
poly.drug				0.49
drug.freq.days				0.40
drug.freq.times				0.12
inject.used.recent	-3.22	(-5.19 ; -1.25)	(-5.33 ; -1.11)	0.57
inject.used.ever				0.07
get.unused.syr				0.02
overdose				0.04
MHI5	-3.72	(-5.63 ; -1.81)	(-5.77 ; -1.67)	0.66
sex.active				0.14
sell.sex.6m				0.01
pay.sex.6m				0.09
HIV.HC.partner_1				0.05
HIV.HC.partner_2				0.05
HIV.test				0.21
HIV.status				0.18
HIV.care_1				0.03
HIV.care_2	-7.22	(-9.32 ; -5.13)	(-9.51 ; -4.94)	1.00
TB				0.27
HepC.treatment_1	-4.49	(-6.76 ; -2.21)	(-6.92 ; -2.06)	0.59
HepC.treatment_2	-5.68	(-9.69 ; -1.66)	(-10.49 ; -0.86)	0.41
HepC.treatment_3				0.05
HepB.aware				0.12
HepB.vaccine	3.08	(1.07 ; 5.09)	(1.02 ; 5.14)	0.33
incarceration				0.12
med.insurance				0.18
med.care.12m				0.12
detox_1				0.65
detox_2				0.48
drug.treat.problems_1				0.09
drug.treat.problems_2				0.19
med.care.problems	-5.21	(-8.99 ; -1.43)	(-9.05 ; -1.37)	0.45
police.confiscate.syr				0.02
IDU.disclosure.close				0.05
IDU.disclosure.doctor				0.03
IDU.stigma.internal	-4.32	(-6.24 ; -2.39)	(-6.33 ; -2.3)	0.87
IDU.stigma.conscious				0.09
**Adjusted R^2**	**0.34**			

Table S3.6. Forward selection regression using Likelihood Ratio Test (p=0.05)

Variables	Forward selection, LRT (p=0.05)			
	Beta	95% CI (asymptotic)	95% CI (bootstrap)	Inclusion frequency
(Intercept)	77.83	(72.7 ; 82.96)	(72.01 ; 83.65)	1.00
sex	-2.39	(-4.55 ; -0.24)	(-4.38 ; -0.4)	0.55
age.d	-5.04	(-6.82 ; -3.26)	(-6.76 ; -3.32)	1.00
education	3.56	(0.29 ; 6.83)	(-0.32 ; 7.45)	0.52
income.source				0.05
income.level	-4.49	(-6.45 ; -2.52)	(-6.48 ; -2.5)	0.99
living.sit_1				0.30
living.sit_2				0.08
marital				0.07
CAGE				0.46
age.drug.use	-3.18	(-5.11 ; -1.24)	(-5.12 ; -1.24)	0.85
main.drug_1				0.07
main.drug_2				0.10
poly.drug	-3.29	(-5.88 ; -0.7)	(-5.82 ; -0.77)	0.75
drug.freq.days	-1.89	(-3.85 ; 0.07)	(-4.04 ; 0.26)	0.47
drug.freq.times				0.16
inject.used.recent	-2.44	(-4.46 ; -0.42)	(-4.72 ; -0.16)	0.64
inject.used.ever				0.12
get.unused.syr				0.07
overdose				0.08
MHI5	-2.81	(-4.71 ; -0.9)	(-4.86 ; -0.76)	0.75
sex.active				0.29
sell.sex.6m				0.10
pay.sex.6m				0.24
HIV.HC.partner_1				0.18
HIV.HC.partner_2				0.15
HIV.test	3.76	(0.1 ; 7.42)	(-0.07 ; 7.58)	0.46
HIV.status				0.36
HIV.care_1				0.10
HIV.care_2	-7.20	(-9.3 ; -5.1)	(-9.45 ; -4.95)	1.00
TB	-3.83	(-7.33 ; -0.32)	(-6.95 ; -0.71)	0.52
HepC.treatment_1	-4.10	(-6.39 ; -1.8)	(-6.49 ; -1.7)	0.81
HepC.treatment_2	-6.40	(-10.37 ; -2.43)	(-11.26 ; -1.54)	0.70
HepC.treatment_3				0.14
HepB.aware				0.15
HepB.vaccine	2.45	(0.43 ; 4.46)	(0.4 ; 4.49)	0.48
incarceration				0.30
med.insurance				0.32
med.care.12m				0.29
detox_1	-5.48	(-8.35 ; -2.6)	(-8.49 ; -2.47)	0.87
detox_2	-4.04	(-7.2 ; -0.88)	(-6.94 ; -1.14)	0.76
drug.treat.problems_1				0.24
drug.treat.problems_2				0.27
med.care.problems	-5.21	(-8.94 ; -1.48)	(-8.99 ; -1.44)	0.64
police.confiscate.syr				0.07
IDU.disclosure.close				0.11
IDU.disclosure.doctor				0.08
IDU.stigma.internal	-3.44	(-5.41 ; -1.47)	(-5.45 ; -1.44)	0.94
IDU.stigma.conscious				0.28
**Adjusted R^2**	**0.37**			
