# Supplementary material for: Comparison of subset selection methods in linear regression in the context of health-related quality of life and substance abuse in Russia
Source: BMC Med Res Methodol. 2015 Aug 30;15:71. doi: 10.1186/s12874-015-0066-2 (PMC4553217; doi:10.1186/s12874-015-0066-2)

Additional file 4: Distribution of the dependent variable

Dependent Variable: EuroQoL 5D visual analogue scale measure of health-related quality of life

Mean = 63.97 Range = (5 – 100)
Median = 65.00 Standard deviation = 15.34

Figure S4.1. Histogram of the dependent variable

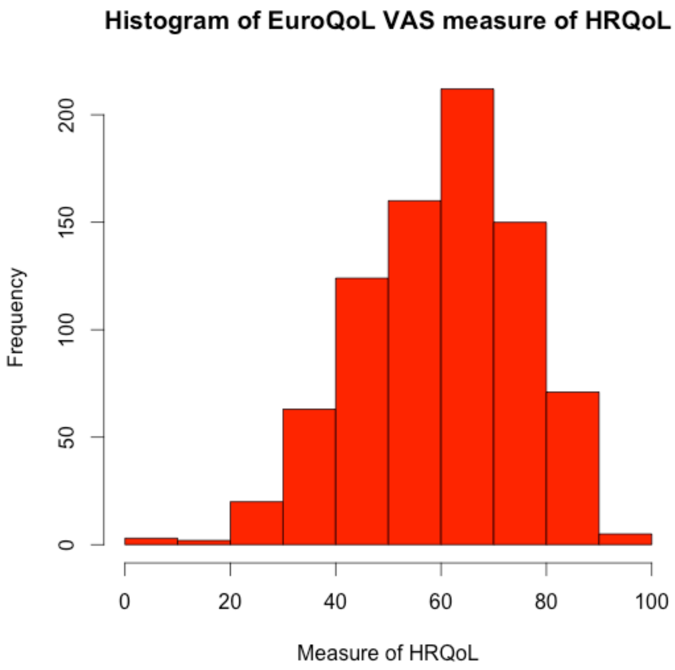

Figure S4.2. Q-Q plot of the transformed (standardized) dependent variable

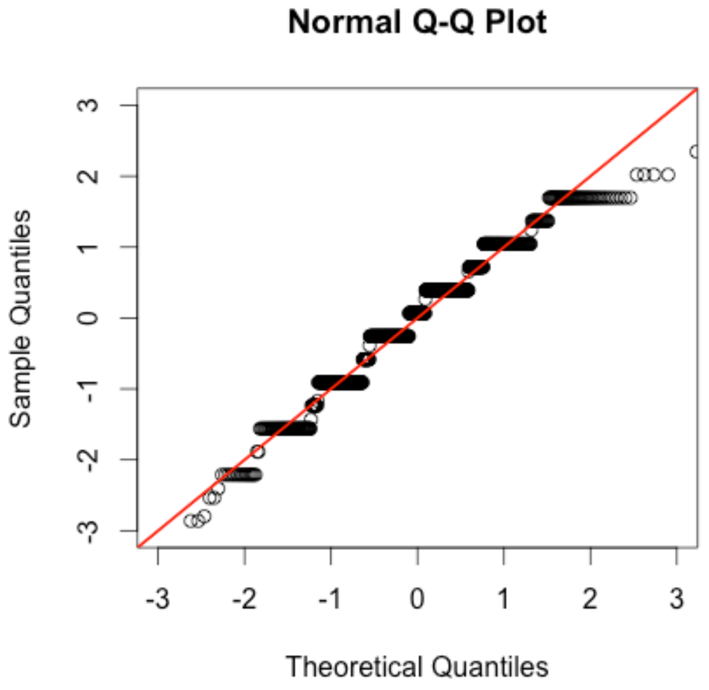

Supplement: Additional file 4: — Distribution of the dependent variable. Descriptive statistics, histogram and Q-Q plot of the dependent variable. (PDF 80 kb) [file 12874_2015_66_MOESM4_ESM.pdf]
