# Supplementary material for: Comparison of subset selection methods in linear regression in the context of health-related quality of life and substance abuse in Russia
Source: BMC Med Res Methodol. 2015 Aug 30;15:71. doi: 10.1186/s12874-015-0066-2 (PMC4553217; doi:10.1186/s12874-015-0066-2)
Supplement: Additional file 5: — Correlation matrix. Matrix of all pairwise correlation coefficients of independent variables considered in subset selection. (PDF 64 kb) [file 12874_2015_66_MOESM5_ESM.pdf]

Additional file 5: Correlation matrix

Variables	sex	age.d	education	income.source	income.level	living.sit_1	living.sit_2	marital	CAGE	age.drug.use	main.drug_1	main.drug_2	poly.drug	drug.freq.days	drug.freq.times	inject.used.recent	inject.used.ever	get.unused.syr	overdose	MHI5	sex.active	sell.sex.6m	pay.sex.6m	HIV.HC.partner_1	HIV.HC.partner_2	HIV.test	HIV.status	HIV.care_1	HIV.care_2	TB	HepC.treatment_1	HepC.treatment_2	HepC.treatment_3	HepB.aware	HepB.vaccine	incarceration	med.insurance	med.care.12m	detox_1	detox_2	drug.treat.problems_1	drug.treat.problems_2	med.care.problems	police.confiscate.syr	IDU.disclosure.close	IDU.disclosure.doctor	IDU.stigma.internal	IDU.stigma.conscious
sex	1.00	-0.25	0.05	0.12	-0.01	0.00	-0.06	0.06	-0.10	-0.11	0.04	-0.08	0.00	-0.05	-0.04	-0.15	-0.15	0.11	-0.18	-0.01	0.05	0.43	-0.12	0.02	-0.06	0.03	-0.05	0.03	-0.06	-0.07	-0.07	-0.01	-0.05	-0.18	0.09	-0.14	-0.01	-0.09	-0.10	0.02	-0.13	0.01	-0.02	-0.15	-0.01	-0.03	0.02	0.01
age.d		1.00	0.02	-0.10	-0.03	0.06	-0.01	0.10	0.09	-0.02	0.05	0.01	-0.13	-0.01	-0.03	0.05	0.07	-0.11	0.14	0.01	-0.09	-0.09	-0.04	0.07	-0.04	0.02	0.08	0.00	0.07	0.04	0.06	0.01	0.00	0.14	-0.04	0.13	0.06	0.04	0.09	-0.05	0.14	-0.11	0.01	0.14	0.01	0.08	-0.01	-0.10
education			1.00	-0.08	-0.08	-0.04	-0.19	0.13	-0.09	-0.13	0.03	-0.03	-0.11	-0.04	-0.13	-0.04	-0.02	-0.03	-0.01	-0.09	0.02	0.02	0.00	0.05	-0.05	0.04	-0.05	0.01	-0.08	-0.04	-0.05	0.05	0.05	-0.08	0.13	-0.13	0.02	0.02	0.05	-0.03	0.08	-0.05	0.03	0.12	-0.06	0.17	-0.01	-0.03
income.source				1.00	0.08	0.01	0.00	-0.12	0.04	0.12	-0.04	0.07	0.09	0.22	0.21	0.10	0.12	0.08	0.09	0.19	0.01	0.32	0.02	-0.02	0.12	0.07	0.10	0.04	0.10	0.04	0.11	0.03	-0.06	0.03	0.06	0.09	-0.17	-0.02	-0.15	0.06	-0.04	0.12	-0.01	-0.04	-0.01	-0.12	-0.07	0.15
income.level					1.00	-0.11	0.07	-0.08	0.20	0.11	-0.14	0.18	0.02	0.15	0.13	0.23	0.15	-0.09	0.11	0.15	-0.10	0.05	-0.11	0.09	0.08	-0.05	0.14	0.00	0.16	0.04	0.10	-0.04	-0.03	0.17	-0.21	0.03	0.01	-0.01	0.05	-0.02	0.06	0.07	0.04	0.08	0.02	-0.16	0.12	0.02
living.sit_1						1.00	-0.24	0.31	0.10	0.09	-0.10	0.12	0.14	0.12	0.21	0.14	0.06	-0.09	0.09	0.21	0.17	0.00	-0.07	0.13	-0.17	0.10	0.02	-0.09	0.15	-0.02	0.10	-0.05	-0.07	0.15	-0.10	-0.04	0.07	-0.06	0.17	0.03	-0.02	0.08	-0.05	0.10	0.30	-0.08	0.23	-0.01
living.sit_2							1.00	-0.11	0.07	-0.01	0.00	0.01	-0.03	-0.07	-0.04	0.01	-0.01	0.01	-0.06	-0.01	-0.11	-0.02	0.00	-0.06	0.06	-0.13	-0.03	-0.02	0.00	0.04	-0.02	0.01	-0.04	-0.04	-0.06	0.12	-0.19	0.09	-0.02	-0.03	-0.13	0.02	0.02	-0.06	0.01	0.05	-0.05	0.01
marital								1.00	-0.03	-0.05	-0.02	0.04	-0.05	-0.05	-0.08	0.00	-0.02	-0.07	0.05	-0.03	0.28	-0.08	-0.16	0.41	-0.56	0.04	-0.04	-0.05	0.02	0.00	-0.04	-0.06	-0.01	0.01	0.03	-0.07	0.09	0.05	0.17	-0.01	0.01	-0.02	-0.04	0.13	0.04	0.12	0.10	-0.06
CAGE									1.00	0.22	-0.21	0.27	0.12	0.19	0.25	0.29	0.20	-0.10	0.26	0.30	0.00	0.04	-0.12	0.06	0.01	0.03	0.10	-0.11	0.26	0.02	0.19	-0.07	-0.03	0.35	-0.17	0.02	0.00	-0.01	0.19	-0.03	0.07	0.13	0.09	0.14	0.25	-0.11	0.23	0.02
age.drug.use										1.00	-0.17	0.23	0.07	0.28	0.26	0.18	0.20	-0.11	0.10	0.24	0.06	0.09	0.02	0.06	0.02	0.03	0.03	-0.08	0.13	-0.03	0.12	-0.06	0.03	0.23	0.07	0.00	-0.06	-0.04	0.02	-0.01	0.03	0.14	0.07	0.07	0.06	-0.04	0.00	0.10
main.drug_1											1.00	-0.92	-0.16	-0.40	-0.36	-0.24	-0.05	0.14	-0.05	-0.22	-0.05	-0.03	0.13	-0.07	0.01	0.01	-0.08	0.06	-0.16	0.03	-0.11	0.14	0.08	-0.26	0.12	0.07	-0.04	-0.03	-0.20	0.02	-0.01	-0.05	0.02	-0.20	-0.22	0.08	-0.21	0.00
main.drug_2												1.00	0.13	0.46	0.39	0.29	0.18	-0.15	0.12	0.27	0.04	0.04	-0.13	0.10	0.00	0.02	0.13	-0.06	0.21	-0.02	0.19	-0.13	-0.06	0.32	-0.12	-0.02	0.02	0.06	0.22	-0.01	0.06	0.06	-0.01	0.24	0.21	-0.07	0.22	0.02
poly.drug													1.00	0.15	0.22	0.09	-0.02	0.04	0.02	0.14	0.08	-0.05	-0.02	0.01	0.01	-0.03	0.02	-0.06	0.09	0.07	0.12	-0.06	-0.08	0.13	-0.06	0.05	0.09	-0.11	-0.12	-0.03	-0.11	0.15	0.01	-0.13	0.20	-0.29	0.16	0.14
drug.freq.days														1.00	0.59	0.34	0.26	-0.04	0.19	0.30	0.04	0.14	-0.01	0.11	0.08	0.01	0.20	0.03	0.22	0.09	0.23	-0.03	-0.01	0.23	0.00	0.03	-0.07	0.02	0.07	0.00	0.02	0.16	0.07	0.19	0.13	-0.10	0.09	0.21
drug.freq.times															1.00	0.28	0.19	-0.08	0.18	0.32	0.03	0.10	-0.07	0.05	0.10	0.03	0.15	-0.02	0.22	0.06	0.18	-0.02	-0.05	0.24	-0.10	0.02	-0.01	-0.05	0.01	0.03	-0.02	0.18	0.09	0.12	0.19	-0.21	0.10	0.12
inject.used.recent																1.00	0.37	-0.19	0.26	0.25	0.03	0.02	-0.04	0.14	0.07	0.06	0.31	-0.02	0.34	0.05	0.24	-0.03	-0.06	0.32	-0.15	-0.07	0.01	0.10	0.24	-0.02	0.10	0.13	0.09	0.27	0.23	0.00	0.20	0.08
inject.used.ever																	1.00	-0.08	0.27	0.19	-0.03	-0.03	0.05	0.10	0.08	0.07	0.26	0.12	0.16	0.05	0.20	0.01	0.02	0.22	-0.11	0.04	-0.04	0.06	0.08	0.02	0.09	0.07	0.03	0.15	0.02	-0.01	0.08	0.11
get.unused.syr																		1.00	-0.12	-0.09	-0.03	0.02	0.07	-0.07	0.01	0.02	-0.10	0.10	-0.13	0.00	-0.08	0.06	0.06	-0.18	0.13	0.10	-0.07	-0.07	-0.30	0.03	-0.09	0.09	0.06	-0.29	-0.18	-0.13	-0.18	0.11
overdose																			1.00	0.14	-0.03	-0.05	-0.07	0.13	-0.05	0.08	0.15	0.00	0.16	0.08	0.21	-0.02	-0.07	0.33	-0.20	0.02	0.07	0.08	0.21	0.04	0.17	0.06	0.00	0.19	0.12	-0.05	0.12	-0.01
MHI5																				1.00	0.04	0.13	-0.11	0.09	0.07	0.04	0.16	-0.05	0.22	0.06	0.16	-0.03	-0.08	0.21	0.01	-0.03	-0.05	-0.05	0.10	0.02	0.00	0.20	0.05	0.13	0.24	-0.10	0.25	0.18
sex.active																					1.00	0.15	0.16	0.33	-0.51	0.04	-0.03	-0.01	0.02	0.02	-0.07	0.03	0.01	0.03	0.11	0.02	0.05	0.04	0.05	0.01	-0.01	0.03	0.04	0.08	-0.02	-0.01	0.03	0.00
sell.sex.6m																						1.00	-0.04	-0.05	0.10	0.01	0.01	0.04	0.00	-0.07	0.01	0.01	0.00	-0.06	0.18	-0.11	-0.20	0.09	-0.02	0.01	-0.08	0.00	0.10	0.07	0.01	0.03	-0.08	0.08
pay.sex.6m																							1.00	-0.14	0.19	0.00	-0.03	0.06	-0.11	0.02	-0.07	0.08	0.03	-0.10	0.16	0.11	-0.06	0.02	-0.09	0.03	0.03	-0.08	0.10	0.02	-0.14	0.08	-0.19	0.05
HIV.HC.partner_1																								1.00	-0.66	0.10	0.16	0.07	0.17	0.07	0.12	0.00	-0.02	0.16	-0.03	0.01	0.06	0.01	0.09	0.01	0.08	0.07	-0.01	0.08	-0.05	0.00	0.14	0.04
HIV.HC.partner_2																									1.00	0.00	0.08	0.03	0.03	0.02	0.07	0.02	0.00	-0.03	-0.02	0.02	-0.14	-0.02	-0.05	-0.02	-0.01	0.03	0.08	-0.02	0.08	-0.03	-0.07	0.06
HIV.test																										1.00	0.15	0.11	0.18	0.07	0.15	0.05	0.02	0.14	0.10	0.10	-0.01	-0.08	0.03	0.04	0.16	0.06	0.04	0.05	0.00	0.07	0.10	0.04
HIV.status																											1.00	0.38	0.61	0.14	0.14	0.01	0.02	0.20	-0.15	0.12	-0.06	0.02	0.03	0.01	0.07	0.14	0.07	0.11	0.03	-0.12	0.12	0.16
HIV.care_1																												1.00	-0.29	0.05	-0.17	0.18	0.22	-0.08	0.13	0.05	0.05	-0.27	-0.14	0.09	0.08	0.02	0.13	-0.05	-0.21	-0.02	-0.17	0.06
HIV.care_2																													1.00	0.13	0.28	-0.09	-0.14	0.31	-0.23	0.12	-0.08	0.13	0.14	-0.05	0.02	0.19	-0.02	0.12	0.24	-0.14	0.26	0.13
TB																														1.00	0.04	0.04	-0.02	0.00	-0.01	0.13	-0.05	0.00	-0.07	0.01	-0.06	0.11	0.10	0.03	-0.01	-0.05	0.04	0.14
HepC.treatment_1																															1.00	-0.42	-0.39	0.32	-0.14	0.05	0.03	0.05	0.13	0.07	0.15	0.08	-0.04	0.09	0.15	-0.11	0.16	0.07
HepC.treatment_2																																1.00	-0.06	-0.07	0.12	0.03	-0.10	-0.04	-0.10	-0.02	-0.01	0.04	0.09	-0.03	-0.16	0.02	-0.07	0.07
HepC.treatment_3																																	1.00	-0.10	0.14	-0.01	0.02	-0.09	-0.09	-0.05	-0.03	-0.03	0.06	-0.05	-0.12	0.04	-0.14	0.04
HepB.aware																																		1.00	-0.28	-0.04	0.12	0.00	0.32	-0.04	0.15	0.10	-0.06	0.20	0.29	-0.05	0.30	0.04
HepB.vaccine																																			1.00	-0.03	-0.09	-0.15	-0.16	-0.05	-0.12	-0.03	0.15	-0.03	-0.23	0.26	-0.30	0.16
incarceration																																				1.00	-0.10	0.03	-0.14	0.03	-0.05	0.12	0.06	-0.12	-0.12	-0.10	-0.12	0.05
med.insurance																																					1.00	-0.18	0.07	-0.02	0.10	-0.06	-0.09	-0.04	0.02	-0.10	0.09	-0.10
med.care.12m																																						1.00	0.19	-0.14	0.03	-0.13	-0.02	0.16	0.04	0.11	0.05	-0.04
detox_1																																							1.00	-0.11	0.20	-0.10	-0.08	0.39	0.32	0.22	0.29	-0.15
detox_2																																								1.00	0.13	0.03	0.00	-0.03	-0.07	-0.13	0.01	-0.03
drug.treat.problems_1																																									1.00	-0.44	-0.06	0.21	0.01	0.04	0.11	-0.12
drug.treat.problems_2																																										1.00	0.21	-0.10	0.04	-0.22	0.06	0.20
med.care.problems																																											1.00	0.11	-0.05	-0.02	-0.10	0.11
police.confiscate.syr																																												1.00	0.16	0.27	0.17	-0.06
IDU.disclosure.close																																													1.00	0.03	0.42	-0.02
IDU.disclosure.doctor																																														1.00	-0.12	-0.14
IDU.stigma.internal																																															1.00	0.11
IDU.stigma.conscious																																																1.00
