# Supplementary material for: Comparison of subset selection methods in linear regression in the context of health-related quality of life and substance abuse in Russia
Source: BMC Med Res Methodol. 2015 Aug 30;15:71. doi: 10.1186/s12874-015-0066-2 (PMC4553217; doi:10.1186/s12874-015-0066-2)
Supplement: Additional file 6: — Penalized regression methods. Detailed outputs of lasso, adaptive lasso, and adaptive elastic net regressions; includes regularization paths, graphs of cross validation results, estimates of regression coefficients along with 95 % CIs, and the bootstrap inclusion frequencies for independent variables. (PDF 920 kb) [file 12874_2015_66_MOESM6_ESM.pdf]

Additional file 6: Penalized regression methods

Note: in the figures two vertical lines represent the following values of λ: a) the value that minimizes the cross-validation MSE (λ_{min}); b) the maximum value within one standard error of the λ_{min} (λ_{1SE}). The numbers at the top axis show the number of non-zero regression coefficients for every value of λ; which corresponds to the number of variables retained in the model.

Figure S6.1: Lasso coefficients path and results of cross-validation

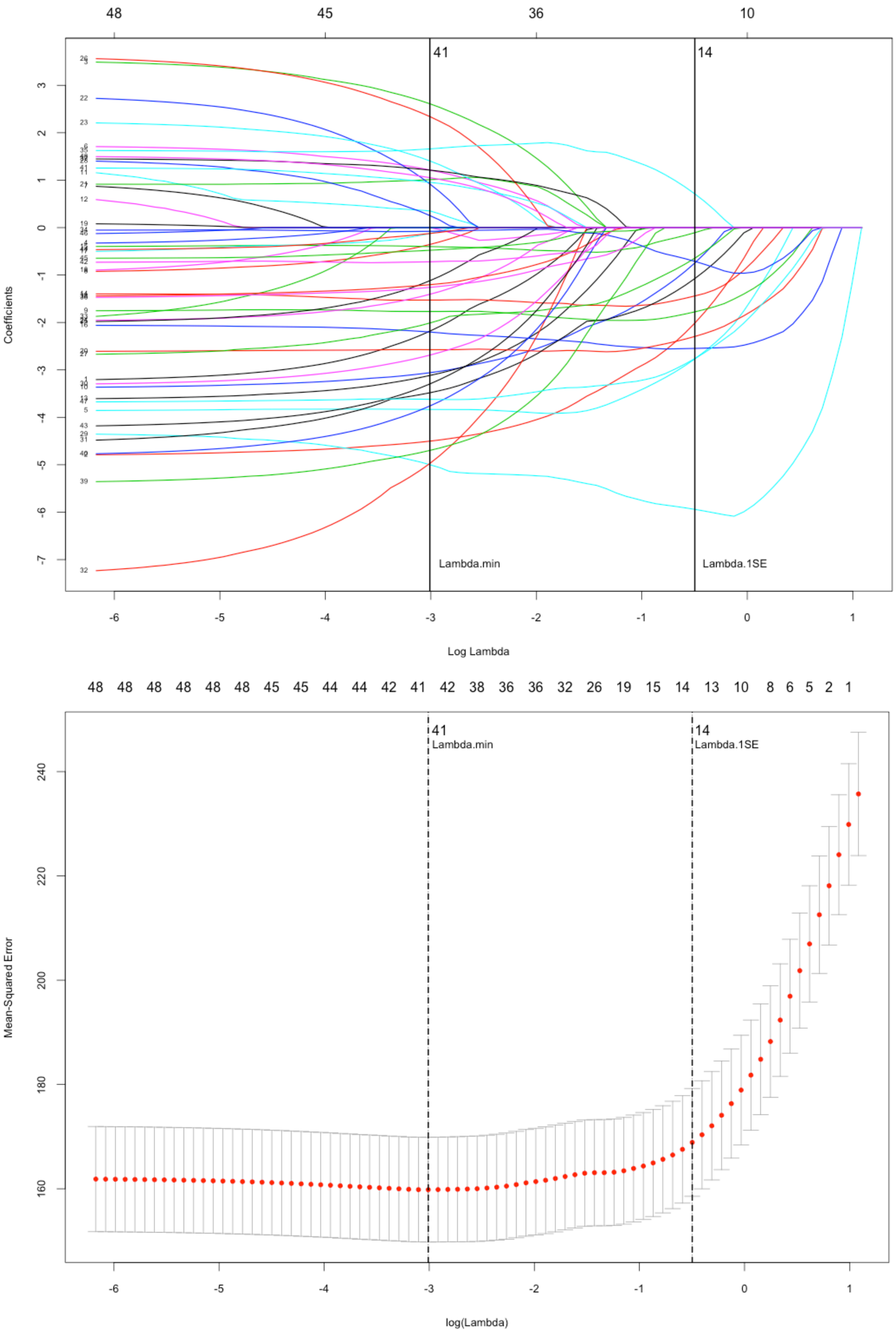

Table S6.1: Lasso coefficients, 95% confidence intervals and inclusion probabilities

Note: Inclusion frequency is estimated using bootstrap.

Variables	Lasso: λ_{min}			Lasso: λ_{1SE}		
	Beta	95% CI	Inclusion frequency	Beta	95% CI	Inclusion frequency
(Intercept)	78.40	(70.76 ; 86.04)	1.00	76.53	(73.89 ; 79.18)	1.00
sex	-2.18	(-4.34 ; -0.03)	0.96			0.20
age.d	-4.50	(-6.29 ; -2.72)	1.00	-2.01	(-3.76 ; -0.26)	1.00
education	2.61	(-1.06 ; 6.28)	0.89			0.30
income.source			0.64			0.05
income.level	-3.83	(-5.93 ; -1.74)	1.00	-2.77	(-4.81 ; -0.73)	1.00
living.sit_1	1.21	(-0.59 ; 3.01)	0.90			0.18
living.sit_2			0.39			0.00
marital	-0.36	(-2.19 ; 1.48)	0.71			0.06
CAGE	-1.77	(-3.85 ; 0.31)	0.95	-1.78	(-3.82 ; 0.27)	0.94
age.drug.use	-3.06	(-5.02 ; -1.09)	1.00	-0.75	(-2.32 ; 0.83)	0.93
main.drug_1	0.35	(-1.26 ; 1.96)	0.53			0.09
main.drug_2			0.51			0.39
poly.drug	-3.12	(-5.84 ; -0.39)	0.99			0.50
drug.freq.days	-1.53	(-3.6 ; 0.54)	0.89	-1.44	(-3.28 ; 0.4)	0.86
drug.freq.times	-0.40	(-2.1 ; 1.3)	0.73	-0.11	(-1.33 ; 1.11)	0.55
inject.used.recent	-2.20	(-4.43 ; 0.03)	0.97	-2.55	(-4.8 ; -0.3)	0.98
inject.used.ever	-0.13	(-2.48 ; 2.23)	0.61			0.07
get.unused.syr			0.52			0.01
overdose			0.74			0.32
MHI5	-2.57	(-4.72 ; -0.42)	0.99	-2.30	(-4.43 ; -0.16)	0.98
sex.active	1.01	(-1.19 ; 3.21)	0.81			0.29
sell.sex.6m	0.92	(-1.73 ; 3.57)	0.58			0.00
pay.sex.6m	1.41	(-1.25 ; 4.06)	0.79			0.10
HIV.HC.partner_1	-1.41	(-3.98 ; 1.17)	0.85			0.29
HIV.HC.partner_2	-1.12	(-3.47 ; 1.23)	0.82			0.26
HIV.test	2.34	(-1.21 ; 5.9)	0.82			0.07
HIV.status	-2.01	(-5.06 ; 1.04)	0.94	-0.65	(-2.45 ; 1.16)	0.85
HIV.care_1	0.25	(-2.74 ; 3.25)	0.60			0.03
HIV.care_2	-5.00	(-8.36 ; -1.64)	0.99	-5.94	(-8.47 ; -3.41)	1.00
TB	-2.69	(-5.57 ; 0.2)	0.94			0.23
HepC.treatment_1	-3.48	(-6.12 ; -0.85)	0.99	-1.07	(-2.99 ; 0.84)	0.90
HepC.treatment_2	-4.97	(-9.98 ; 0.03)	0.95			0.16
HepC.treatment_3			0.44			0.01
HepB.aware	-0.08	(-1.81 ; 1.65)	0.72	-0.71	(-2.36 ; 0.94)	0.58
HepB.vaccine	1.66	(-0.46 ; 3.78)	0.93	0.71	(-0.84 ; 2.27)	0.84
incarceration	-1.26	(-3 ; 0.48)	0.92			0.53
med.insurance	1.21	(-1.07 ; 3.49)	0.85			0.38
med.care.12m	-1.20	(-3 ; 0.59)	0.89			0.40
detox_1	-4.70	(-8.16 ; -1.24)	0.99			0.60
detox_2	-3.76	(-6.85 ; -0.67)	0.98			0.29
drug.treat.problems_1	0.95	(-0.87 ; 2.78)	0.84			0.20
drug.treat.problems_2	-0.72	(-3.39 ; 1.94)	0.71			0.30
med.care.problems	-3.30	(-6.94 ; 0.35)	0.93			0.22
police.confiscate.syr	-0.12	(-2.06 ; 1.81)	0.72			0.19
IDU.disclosure.close	-0.48	(-2.23 ; 1.28)	0.79			0.46
IDU.disclosure.doctor			0.72			0.19
IDU.stigma.internal	-3.62	(-5.78 ; -1.46)	1.00	-2.76	(-4.8 ; -0.72)	1.00
IDU.stigma.conscious	1.05	(-0.79 ; 2.89)	0.87			0.18

Figure S6.2: Adaptive lasso coefficients path and results of cross-validation

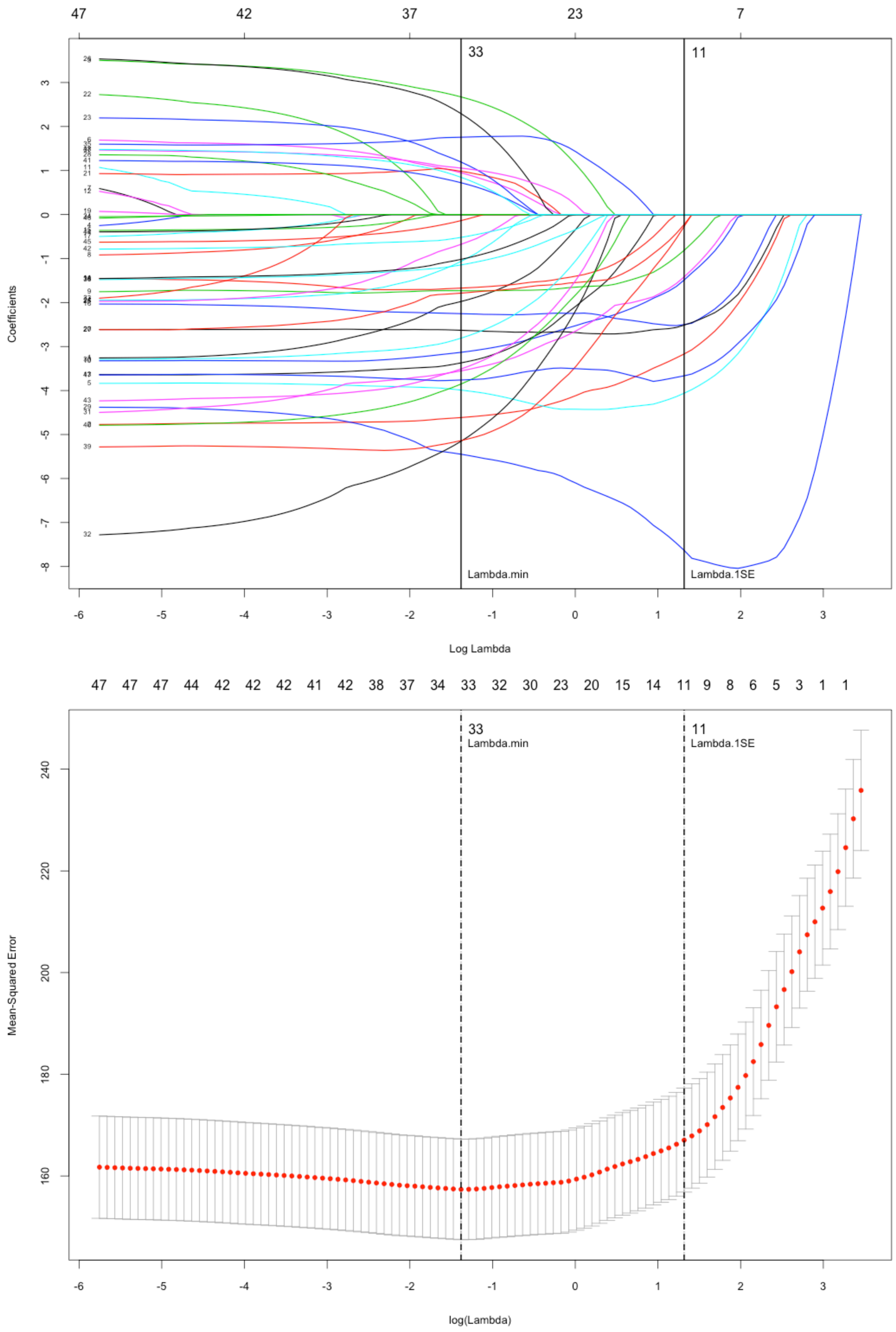

Table S6.2: Adaptive lasso coefficients, 95% confidence intervals and inclusion probabilities

Note: Inclusion frequency is estimated using bootstrap

Variables	Adaptive Lasso: λ_{min}			Adaptive Lasso: λ_{1SE}		
	Beta	95% CI	Inclusion frequency	Beta	95% CI	Inclusion frequency
(Intercept)	78.58	(71.93 ; 85.23)	1.00	77.98	(75.61 ; 80.35)	1.00
sex	-1.98	(-3.96 ; 0.01)	0.89			0.20
age.d	-4.61	(-6.36 ; -2.86)	1.00	-3.17	(-4.93 ; -1.4)	1.00
education	2.67	(-0.92 ; 6.27)	0.80			0.38
income.source			0.32			0.02
income.level	-3.99	(-6.05 ; -1.93)	1.00	-4.06	(-6.07 ; -2.05)	0.99
living.sit_1	0.95	(-0.71 ; 2.61)	0.73			0.12
living.sit_2			0.24			0.01
marital			0.45			0.04
CAGE	-1.73	(-3.77 ; 0.31)	0.87	-0.81	(-2.54 ; 0.91)	0.63
age.drug.use	-3.13	(-5.06 ; -1.2)	0.99	-1.49	(-3.28 ; 0.3)	0.84
main.drug_1			0.32			0.06
main.drug_2			0.29			0.06
poly.drug	-3.37	(-6.02 ; -0.72)	0.95			0.55
drug.freq.days	-1.67	(-3.66 ; 0.32)	0.77			0.51
drug.freq.times			0.45			0.17
inject.used.recent	-2.25	(-4.48 ; -0.03)	0.91	-2.50	(-4.68 ; -0.32)	0.75
inject.used.ever			0.37			0.06
get.unused.syr			0.31			0.01
overdose			0.39			0.06
MHI5	-2.63	(-4.75 ; -0.52)	0.96	-2.51	(-4.59 ; -0.44)	0.84
sex.active	0.98	(-1.04 ; 3)	0.60			0.15
sell.sex.6m			0.41			0.01
pay.sex.6m	1.19	(-1.26 ; 3.64)	0.64			0.10
HIV.HC.partner_1	-1.07	(-3.45 ; 1.32)	0.70			0.24
HIV.HC.partner_2	-0.68	(-2.73 ; 1.37)	0.66			0.21
HIV.test	2.29	(-1.13 ; 5.72)	0.71			0.14
HIV.status	-1.79	(-4.53 ; 0.96)	0.87	-0.23	(-1.74 ; 1.29)	0.61
HIV.care_1			0.40			0.05
HIV.care_2	-5.45	(-8.52 ; -2.37)	0.98	-7.63	(-10.08 ; -5.19)	0.98
TB	-2.81	(-5.7 ; 0.08)	0.86			0.28
HepC.treatment_1	-3.55	(-5.92 ; -1.19)	0.97	-1.42	(-3.42 ; 0.59)	0.81
HepC.treatment_2	-5.15	(-9.93 ; -0.38)	0.92			0.39
HepC.treatment_3			0.27			0.01
HepB.aware			0.43			0.14
HepB.vaccine	1.76	(-0.26 ; 3.77)	0.79			0.47
incarceration	-1.15	(-2.83 ; 0.54)	0.76			0.27
med.insurance	1.05	(-1.05 ; 3.16)	0.67			0.22
med.care.12m	-1.02	(-2.72 ; 0.68)	0.69			0.17
detox_1	-5.14	(-8.43 ; -1.85)	0.98	-0.27	(-2.26 ; 1.72)	0.72
detox_2	-3.85	(-6.89 ; -0.81)	0.94			0.44
drug.treat.problems_1	0.72	(-0.83 ; 2.27)	0.59			0.11
drug.treat.problems_2	-0.52	(-2.86 ; 1.82)	0.53			0.18
med.care.problems	-3.51	(-7.12 ; 0.1)	0.87			0.35
police.confiscate.syr			0.44			0.08
IDU.disclosure.close	-0.14	(-1.54 ; 1.26)	0.50			0.14
IDU.disclosure.doctor			0.39			0.06
IDU.stigma.internal	-3.76	(-5.87 ; -1.65)	1.00	-3.67	(-5.68 ; -1.65)	0.97
IDU.stigma.conscious	0.85	(-0.84 ; 2.53)	0.67			0.12

Figure S6.3: Adaptive elastic net coefficients path and results of cross-validation

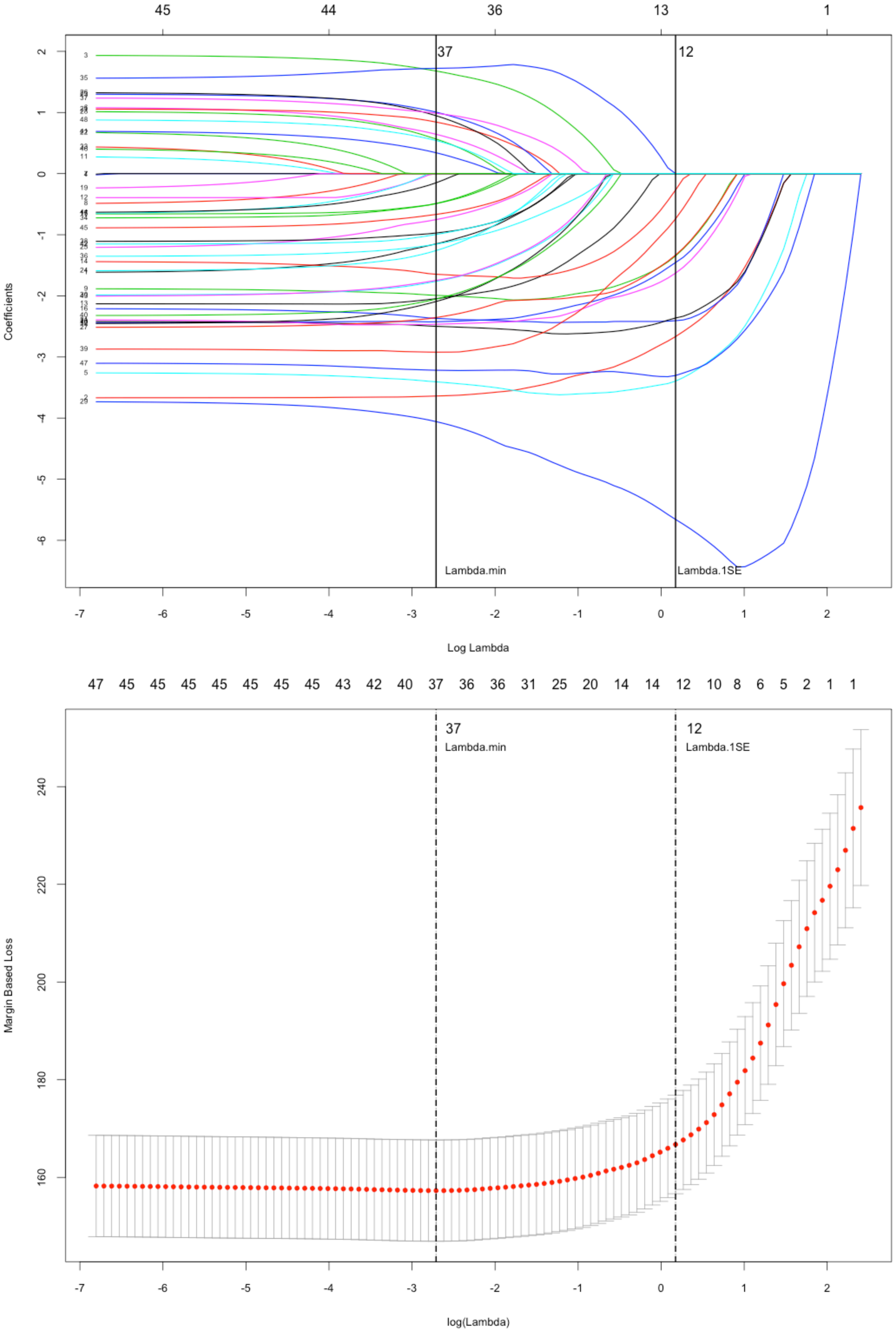

Table S6.3: Adaptive elastic net coefficients, 95% confidence intervals and inclusion probabilities

Note: Inclusion frequency is estimated using bootstrap

Variables	Adaptive Elastic Net: λ_{min}			Adaptive Elastic Net: λ_{1SE}		
	Beta	95% CI	Inclusion frequency	Beta	95% CI	Inclusion frequency
(Intercept)	78.82	(74.27 ; 83.38)	1.00	77.64	(75.51 ; 79.77)	1.00
sex	-1.15	(-2.51 ; 0.21)	0.92			0.21
age.d	-3.64	(-5 ; -2.28)	1.00	-2.67	(-4.1 ; -1.23)	1.00
education	1.68	(-0.31 ; 3.67)	0.88			0.44
income.source			0.52			0.03
income.level	-3.41	(-4.93 ; -1.88)	1.00	-3.40	(-4.96 ; -1.83)	0.99
living.sit_1	0.64	(-0.59 ; 1.88)	0.80			0.14
living.sit_2			0.44			0.01
marital			0.62			0.05
CAGE	-1.99	(-3.53 ; -0.46)	0.96	-1.35	(-2.88 ; 0.19)	0.76
age.drug.use	-2.42	(-3.86 ; -0.99)	1.00	-1.45	(-2.9 ; -0.01)	0.89
main.drug_1			0.52			0.09
main.drug_2			0.49			0.10
poly.drug	-2.05	(-3.73 ; -0.36)	0.98			0.61
drug.freq.days	-1.65	(-3.13 ; -0.17)	0.89	-0.27	(-1.33 ; 0.79)	0.60
drug.freq.times	-0.49	(-1.63 ; 0.65)	0.64			0.23
inject.used.recent	-2.37	(-4 ; -0.74)	0.97	-2.41	(-4.1 ; -0.71)	0.84
inject.used.ever			0.59			0.10
get.unused.syr			0.51			0.02
overdose			0.58			0.09
MHI5	-2.50	(-4.11 ; -0.89)	0.99	-2.36	(-4 ; -0.73)	0.90
sex.active	0.84	(-0.6 ; 2.29)	0.76			0.19
sell.sex.6m			0.57			0.01
pay.sex.6m	1.01	(-0.36 ; 2.37)	0.82			0.16
HIV.HC.partner_1	-1.26	(-2.8 ; 0.28)	0.87			0.35
HIV.HC.partner_2	-0.75	(-2.05 ; 0.54)	0.83			0.26
HIV.test	0.95	(-0.59 ; 2.48)	0.80			0.15
HIV.status	-2.35	(-3.88 ; -0.83)	0.98	-1.33	(-2.84 ; 0.17)	0.81
HIV.care_1	0.57	(-0.69 ; 1.82)	0.69			0.10
HIV.care_2	-4.06	(-5.66 ; -2.45)	1.00	-5.66	(-7.37 ; -3.94)	0.99
TB	-1.76	(-3.22 ; -0.3)	0.95			0.38
HepC.treatment_1	-2.47	(-4.04 ; -0.89)	0.99	-1.63	(-3.24 ; -0.01)	0.88
HepC.treatment_2	-2.09	(-4.12 ; -0.06)	0.96			0.39
HepC.treatment_3			0.47			0.02
HepB.aware	-0.49	(-1.74 ; 0.76)	0.64			0.23
HepB.vaccine	1.72	(0.22 ; 3.22)	0.90			0.57
incarceration	-1.15	(-2.5 ; 0.19)	0.87			0.35
med.insurance	0.99	(-0.62 ; 2.6)	0.80			0.28
med.care.12m	-0.97	(-2.31 ; 0.36)	0.83			0.24
detox_1	-2.92	(-4.79 ; -1.05)	0.99	-0.74	(-2.32 ; 0.84)	0.79
detox_2	-2.05	(-3.72 ; -0.38)	0.97			0.48
drug.treat.problems_1	0.34	(-0.77 ; 1.46)	0.71			0.13
drug.treat.problems_2	-1.00	(-2.59 ; 0.6)	0.73			0.25
med.care.problems	-1.75	(-3.44 ; -0.06)	0.94			0.40
police.confiscate.syr	-0.15	(-1.25 ; 0.95)	0.65			0.14
IDU.disclosure.close	-0.67	(-1.95 ; 0.61)	0.72			0.23
IDU.disclosure.doctor			0.58			0.09
IDU.stigma.internal	-3.22	(-4.73 ; -1.7)	1.00	-3.30	(-4.86 ; -1.74)	0.98
IDU.stigma.conscious	0.54	(-0.72 ; 1.8)	0.76			0.14
