# Supplementary material for: Comparison of subset selection methods in linear regression in the context of health-related quality of life and substance abuse in Russia
Source: BMC Med Res Methodol. 2015 Aug 30;15:71. doi: 10.1186/s12874-015-0066-2 (PMC4553217; doi:10.1186/s12874-015-0066-2)

Additional file 7: Bayesian model averaging

Table S7. BMA results: posterior inclusion probabilities, estimates of regression coefficients and credible intervals

Variables	Aggregate information					100 best models				
	PIP	Unconditional		Conditional		PIP	Unconditional		Conditional	
		Beta	95% CI	Beta	95% CI		Beta	95% CI	Beta	95% CI
sex	0.13					0.00				
age.d	1.00	-4.62	(-6.46 ; -2.78)	-4.62	(-6.46 ; -2.78)	1.00	-4.58	(-6.35 ; -2.81)	-4.58	(-6.35 ; -2.81)
education	0.35					0.19				
income.source	0.04					0.00				
income.level	1.00	-5.03	(-7.09 ; -2.96)	-5.03	(-7.09 ; -2.97)	1.00	-5.05	(-7.09 ; -3.01)	-5.05	(-7.09 ; -3.01)
living.sit_1	0.09					0.00				
living.sit_2	0.05					0.00				
marital	0.04					0.00				
CAGE	0.28					0.00				
age.drug.use	0.81	-2.59	(-5.68 ; 0.49)	-3.21	(-5.24 ; -1.18)	0.99	-3.22	(-5.23 ; -1.2)	-3.24	(-5.19 ; -1.28)
main.drug_1	0.07					0.00				
main.drug_2	0.07					0.00				
poly.drug	0.58	-2.18	(-6.37 ; 2.01)	-3.76	(-6.5 ; -1.02)	0.75	-2.84	(-6.79 ; 1.11)	-3.80	(-6.43 ; -1.17)
drug.freq.days	0.42					0.33				
drug.freq.times	0.14					0.00				
inject.used.recent	0.61	-1.83	(-5.16 ; 1.49)	-3.00	(-5.16 ; -0.85)	0.58	-1.69	(-4.88 ; 1.51)	-2.88	(-4.94 ; -0.83)
inject.used.ever	0.07					0.00				
get.unused.syr	0.04					0.00				
overdose	0.05					0.00				
MHI5	0.84	-2.74	(-5.72 ; 0.25)	-3.25	(-5.29 ; -1.22)	1.00	-3.39	(-5.33 ; -1.44)	-3.39	(-5.33 ; -1.44)
sex.active	0.11					0.00				
sell.sex.6m	0.04					0.00				
pay.sex.6m	0.10					0.00				
HIV.HC.partner_1	0.05					0.00				
HIV.HC.partner_2	0.07					0.00				

Variables	Aggregate information					100 best models				
	PIP	Unconditional		Conditional		PIP	Unconditional		Conditional	
		Beta	95% CI	Beta	95% CI		Beta	95% CI	Beta	95% CI
HIV.test	0.20					0.03				
HIV.status	0.17					0.01				
HIV.care_1	0.05					0.00				
HIV.care_2	1.00	-7.32	(-10.05 ; -4.58)	-7.35	(-9.92 ; -4.78)	1.00	-7.55	(-9.74 ; -5.36)	-7.55	(-9.74 ; -5.36)
TB	0.26					0.09				
HepC.treatment_1	0.77	-3.05	(-7.06 ; 0.96)	-3.96	(-6.62 ; -1.29)	0.91	-3.74	(-7.04 ; -0.44)	-4.13	(-6.55 ; -1.7)
HepC.treatment_2	0.59	-3.51	(-10.12 ; 3.1)	-5.96	(-10.21 ; -1.71)	0.83	-5.03	(-10.8 ; 0.75)	-6.07	(-10.06 ; -2.08)
HepC.treatment_3	0.05					0.00				
HepB.aware	0.05					0.00				
HepB.vaccine	0.39					0.66	1.89	(-1.25 ; 5.02)	2.87	(0.85 ; 4.89)
incarceration	0.13					0.00				
med.insurance	0.16					0.01				
med.care.12m	0.09					0.00				
detox_1	0.81	-3.99	(-8.73 ; 0.75)	-4.93	(-8.09 ; -1.77)	0.90	-4.36	(-8.38 ; -0.33)	-4.83	(-7.85 ; -1.81)
detox_2	0.48					0.42				
drug.treat.problems_1	0.11					0.01				
drug.treat.problems_2	0.14					0.00				
med.care.problems	0.53	-2.71	(-8.5 ; 3.07)	-5.17	(-9.04 ; -1.29)	0.77	-4.21	(-9.85 ; 1.43)	-5.50	(-9.28 ; -1.71)
police.confiscate.syr	0.04					0.00				
IDU.disclosure.close	0.05					0.00				
IDU.disclosure.doctor	0.04					0.00				
IDU.stigma.internal	0.97	-3.94	(-6.48 ; -1.41)	-4.06	(-6.24 ; -1.88)	1.00	-3.71	(-5.78 ; -1.65)	-3.71	(-5.78 ; -1.65)
IDU.stigma.conscious	0.06					0.00				

95% CI, 95% Credible Interval; PIP, posterior inclusion probability.
Conditional: coefficients and 95% CI are estimated by averaging over values conditional on inclusion in the model;
Unconditional: coefficients and 95% CI are estimated by averaging over values from all or 100 best models considered (the coefficient is considered 0 when a model does not include the variable).

Figure S7.1 Posterior model size distribution

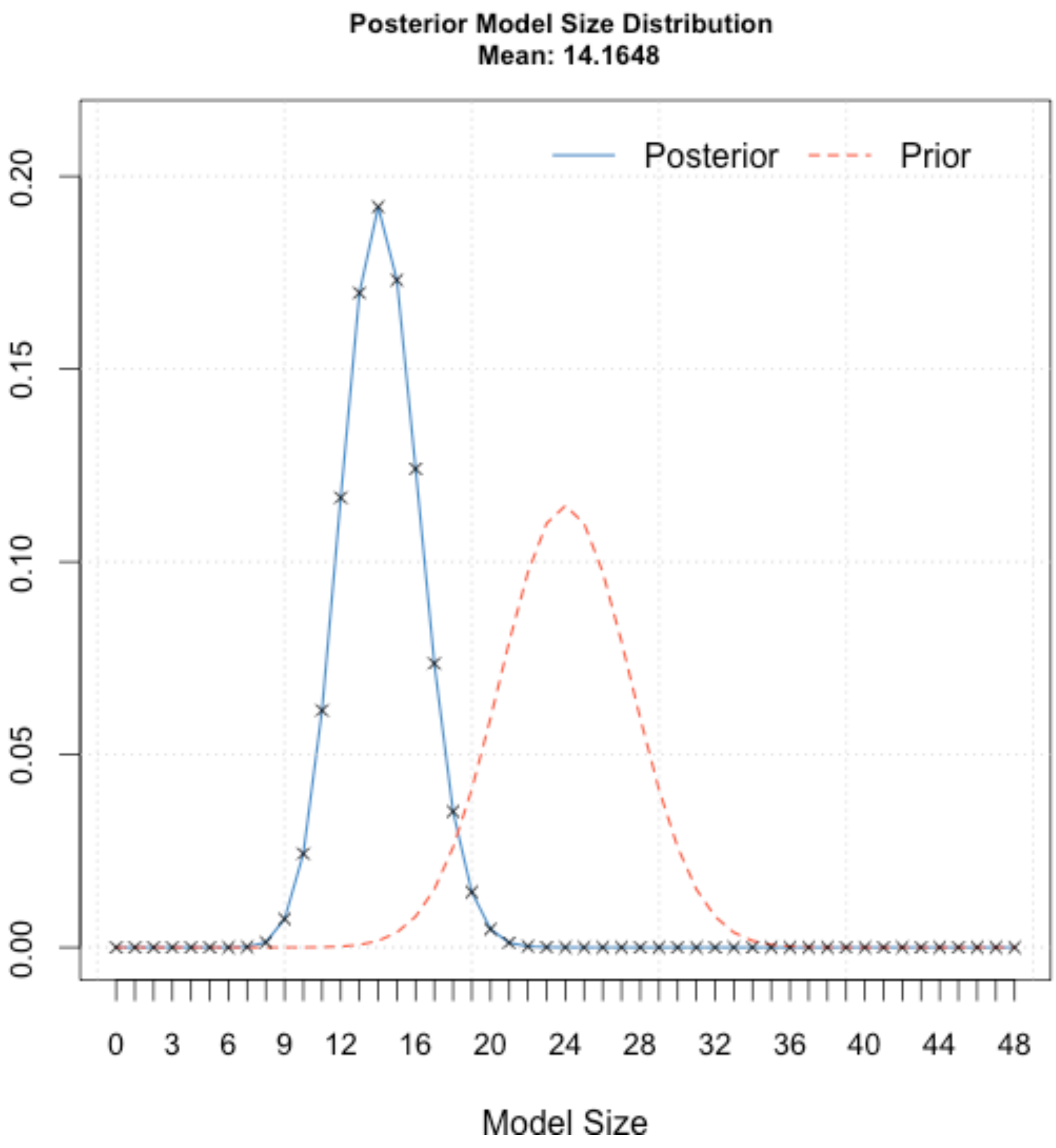

Figure S7.2 Posterior model probabilities (2,000 best subsets) and MCMC chain convergence

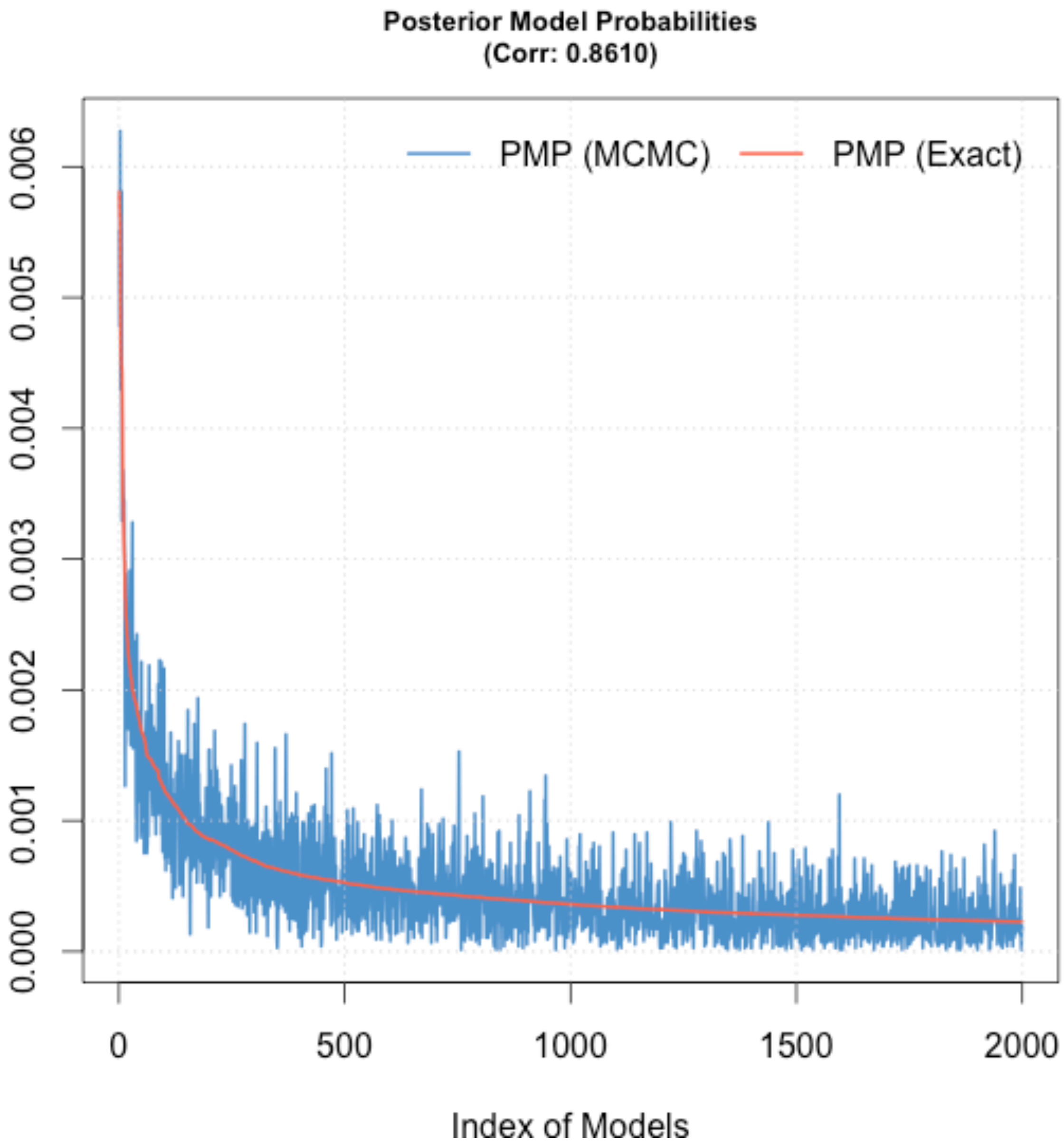

Figure S7.3 Variables inclusion in 2,000 best models evaluated
Note: red color corresponds to negative coefficients, blue – to positive coefficients.

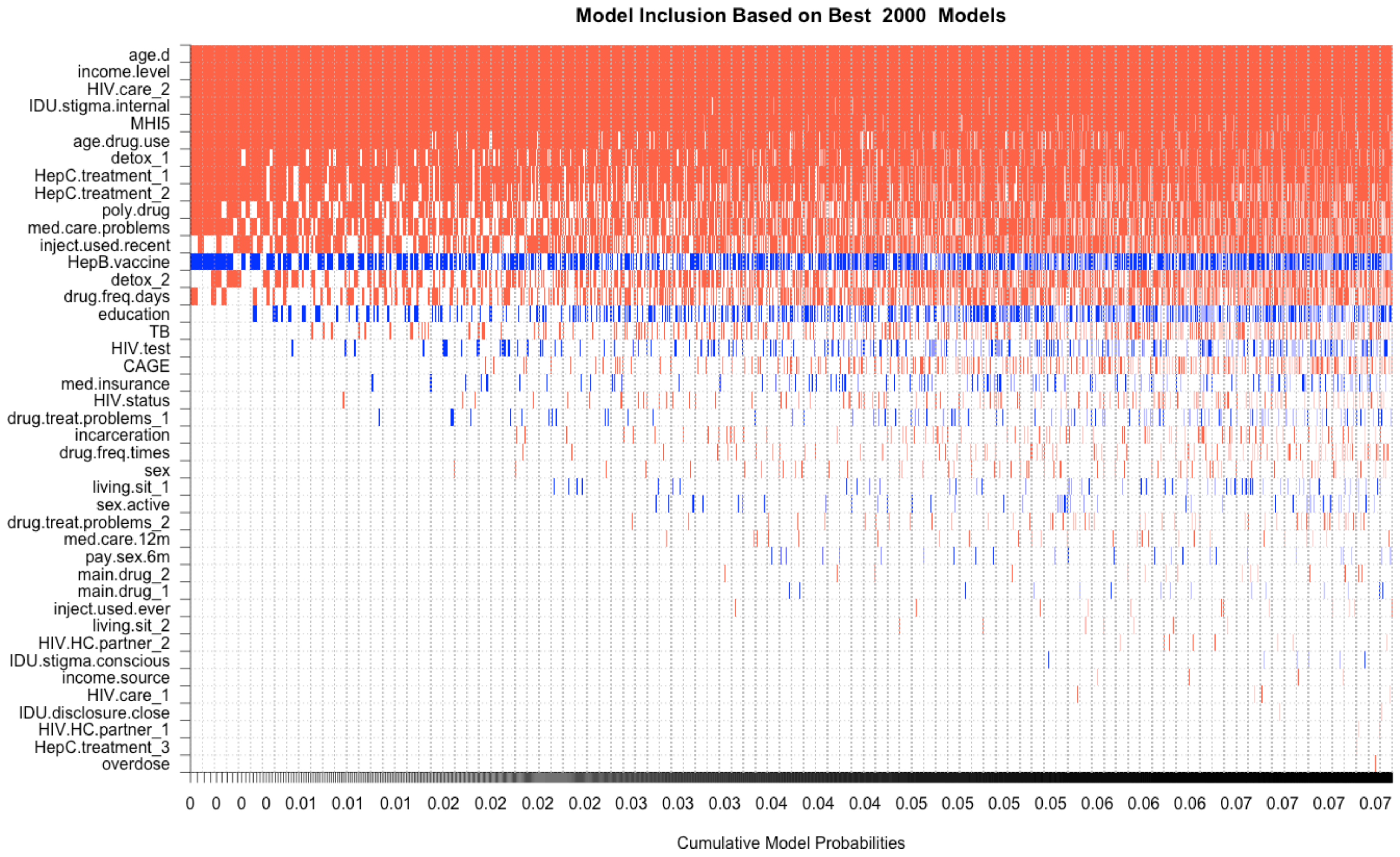

Supplement: Additional file 7: — Bayesian model averaging. BMA posterior inclusion probabilities for model variables, along with regression coefficients and 95 % credible intervals for variables selected in the final model; includes graphs of posterior model size distribution, MCMC chain convergence, and variable inclusion in 2,000 models with the highest likelihood. (PDF 460 kb) [file 12874_2015_66_MOESM7_ESM.pdf]
